# Health-related quality of life and healthcare costs of symptoms and cardiovascular disease events in patients with atrial fibrillation: a longitudinal analysis of 27 countries from the EURObservational Research Programme on Atrial Fibrillation general long-term registry

**DOI:** 10.1093/europace/euae146

**Published:** 2024-05-29

**Authors:** Marjan Walli-Attaei, Mathew Little, Ramon Luengo-Fernandez, Alastair Gray, Aleksandra Torbica, Aldo P Maggioni, Firoozeh Bairami, Radu Huculeci, Victor Aboyans, Adam D Timmis, Panos Vardas, Jose Leal

**Affiliations:** Health Economics Research Centre, Nuffield Department of Population Health, University of Oxford, Oxford, UK; Health Economics Research Centre, Nuffield Department of Population Health, University of Oxford, Oxford, UK; National Perinatal Epidemiology Unit, Nuffield Department of Population Health, University of Oxford, Oxford, UK; Health Economics Research Centre, Nuffield Department of Population Health, University of Oxford, Oxford, UK; Centre for Research on Health and Social Care Management (CERGAS), Bocconi University, Milan, Italy; ANMCO Research Center-Heart Care Foundation, Firenze, Italy; European Heart Agency, European Society of Cardiology, Brussels, Belgium; European Heart Agency, European Society of Cardiology, Brussels, Belgium; Department of Cardiology, Dupuytren University Hospital, EpiMaCT, Inserm1094/IRD270, Limoges University, Limoges, France; William Harvey Research Institute, Queen Mary University London, London UK; Hygeia Hospitals Group, HHG, Biomedical Research Foundation Academy of Athens (BRFAA), Athens, Greece; Health Economics Research Centre, Nuffield Department of Population Health, University of Oxford, Oxford, UK

**Keywords:** Atrial fibrillation, Health-related quality of life, Healthcare costs

## Abstract

**Aims:**

We examine the effects of symptoms and cardiovascular disease (CVD) events on health-related quality of life (HRQOL) and healthcare costs in a European population with atrial fibrillation (AF).

**Methods and results:**

In the EURObservational Research Programme on AF long-term general registry, AF patients from 250 centres in 27 European countries were enrolled and followed for 2 years. We used fixed effects models to estimate the association of symptoms and CVD events on HRQOL and annual healthcare costs. We found significant decrements in HRQOL in AF patients in whom ST-segment elevation myocardial infarction (STEMI) [−0.075 (95% confidence interval −0.144, −0.006)], angina or non-ST-elevation myocardial infarction (NSTEMI) [−0.037 (−0.071, −0.003)], new-onset/worsening heart failure [−0.064 (−0.088, −0.039)], bleeding events [−0.031 (−0.059, −0.003)], thromboembolic events [−0.071 (−0.115, −0.027)], mild symptoms [0.037 (−0.048, −0.026)], or severe/disabling symptoms [−0.090 (−0.108, −0.072)] occurred during the follow-up. During follow-up, annual healthcare costs were associated with an increase of €11 718 (€8497, €14 939) in patients with STEMI, €5823 (€4757, €6889) in patients with angina/NSTEMI, €3689 (€3219, €4158) in patients with new-onset or worsening heart failure, €3792 (€3315, €4270) in patients with bleeding events, and €3182 (€2483, €3881) in patients with thromboembolic events, compared with AF patients without these events. Healthcare costs were primarily driven by inpatient costs. There were no significant differences in HRQOL or healthcare resource use between EU regions or by sex.

**Conclusion:**

Symptoms and CVD events are associated with a high burden on AF patients and healthcare systems throughout Europe.

## Graphical Abstract

**Graphical Abstract:**
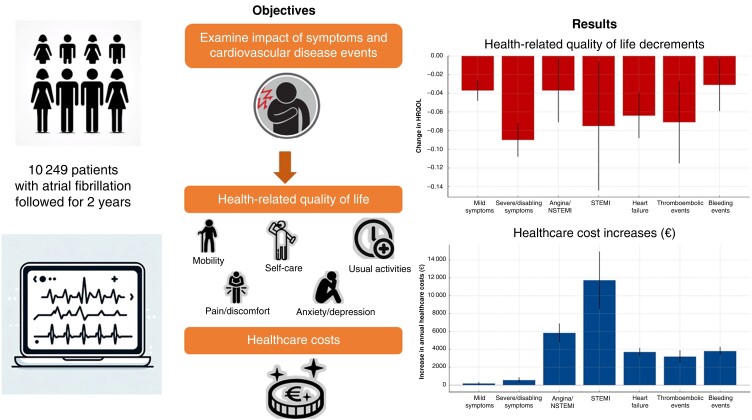

**What’s new?**

- We examine the association of symptoms and cardiovascular disease (CVD) events on health-related quality of life (HRQOL) and healthcare costs in 10 249 atrial fibrillation (AF) patients from 27 European and Central Asian countries followed for 2 years.
- We found that symptoms and CVD events—particularly ST-segment elevation myocardial infarction (STEMI), thromboembolic events, new-onset/worsening heart failure, and severe/disabling symptoms—significantly reduced HRQOL.
- Cardiovascular disease events were associated with significant increases in healthcare costs, with annual cost increases ranging from €3182 for AF patients with thromboembolic events to €11 718 for AF patients with STEMI.
- Our study highlights the significant burden that AF-related symptoms and CVD events place on both patients and healthcare systems across Europe and Central Asia.

## Introduction

Atrial fibrillation (AF) is the most common cardiac arrhythmia, affecting 60 million people worldwide, 14 million of whom live in Europe.^1^ Atrial fibrillation has significant health implications; it is associated with a five-fold increase in the risk of stroke,^2^ a three-fold increase in the risk of heart failure,^3^ and a two-fold increase in the risk of myocardial infarction.^4^ Approximately three-quarters of AF patients exhibit clinical symptoms.^5^ In addition, previous estimates of the average annual cost of managing patients with AF in Europe ranged from €450 to €3000, with marked differences in costs based on patient co-morbidities.^6,7^

Innovations in the treatment and management of AF patients, such as the use of non-vitamin K antagonist oral anticoagulants to prevent stroke and more effective ablation of arrhythmia to reduce AF symptoms, may have improved the quality of life of patients and reduced the costs of AF to healthcare systems.^8^ Therefore, the available evidence from previous studies, many of which enrolled patients prior to changes in guideline recommendations emphasizing stroke prevention and symptom management, may not reflect the current clinical landscape.

It is anticipated that the prevalence of AF will increase due to population aging, improved AF diagnostic methodologies, as well as an increase in cardiovascular risk factors that contribute to the development of AF, such as hypertension and obesity.^1,9,10^ However, a comprehensive assessment in AF populations of the association of symptoms and cardiovascular disease (CVD) events on health-related quality of life (HRQOL) and healthcare costs is currently not available. To address this gap, we used data from a prospectively followed patient population from 27 countries across Europe and Central Asia and estimated the associations of symptoms and CVD events on AF patients’ HRQOL and healthcare costs.

## Methods

### Study design and procedures

The EURObservational Research Programme on Atrial Fibrillation (EORP-AF) long-term general registry is a prospective multinational registry of patients with AF, their management, and clinical outcomes. Details of the study design have been previously described.^11–13^ Briefly, patients at least 18 years of age or older were eligible for enrolment if a 12-lead electrocardiogram or other electrocardiographic documentation revealed an AF event at the baseline visit or within 12 months prior to enrolment. Patients were enrolled from 250 inpatient and outpatient centres in 27 countries and followed for 2 years. The participating countries are listed in the appendix (see Supplementary material online, *Table A1 p 2*). All patients provided written consent, and the study was conducted according to the European Union Note for Guidance on Good Clinical Practice CPMP/ECH/135/95 and the Declaration of Helsinki. This study is reported according to the guidelines for strengthening the reporting of observational studies in epidemiology (see Supplementary material online, *Appendix pp 22–24*).

During the enrolment admission, investigators gathered information from medical records and patient interviews about patients’ characteristics, cardiovascular risk factors, co-morbidities, symptomatic status, HRQOL, medical treatments, and management. At 12- and 24-month follow-up visits, investigators gathered information on patients’ symptomatic status, the occurrence of adverse events, HRQOL, medical treatments and management, and clinical visits for CVD reasons during the year [i.e. emergency room (ER) admissions, cardiology visits, and internal medicine/general practitioner (GP) visits].

### Outcomes

The primary outcomes considered in our analysis were HRQOL and healthcare costs. The secondary outcomes were number of ER admissions, cardiology visits, and internal medicine/GP visits.

Health-related quality of life was measured using the EUROQoL 5-Dimension 5-Level (EQ-5D-5L) questionnaire. The EQ-5D-5L questionnaire consists of questions about five dimensions: mobility, self-care, usual activities, pain or discomfort, and anxiety or depression. Each dimension has five response levels and patients are asked to choose one of the levels for each dimension that reflect their ‘own health state today’, representing ‘no problems’, ‘slight problems’, ‘moderate problems’, ‘severe problems’, and ‘extreme problems’. We followed recommendations by NICE and mapped EQ-5D-5L responses to EQ-5D-3L^14^ and then used the latter value set as there is currently no valid UK value set for EQ-5D-5L.^15^ Health utilities based on the UK value set range from −0.594 to 1, where 1 indicates perfect health and 0 equals death.^14^ In sensitivity analyses, we used value sets from Spain,^16^ Germany,^17^ and Slovenia.^18^

Healthcare costs consisted of inpatient admissions, ER admissions, clinical visits (cardiology and internal medicine/GP visits), and medications (e.g. anticoagulation, antiplatelet agents, CVD, and diabetes medications). The resource utilization in the registry during follow-up was costed. To combine the various types of resource use (e.g. admissions and medications) into a single metric and enable cross-country comparisons of resource volume, we valued all resource use in the same currency, UK NHS costs for 2018/19.^19,20^ Applying unit costs from a single country is often done in multi-country costing^21^ and cost-effectiveness^22^ studies, because it allows for consistency in the evaluation framework, enabling direct comparisons of healthcare resource volume across countries, and increases statistical power.

Our approach to costing assumes that the relative differences in the costs of event-related admissions, diagnostics, and pharmaceuticals in the UK are similar across countries. To test this assumption, we conducted sensitivity analyses using costs from Spain,^23^ Germany,^24^ and Slovenia.^25^ We selected these countries because, like the UK, they all have DRG systems and a robust costing framework that regularly produces and updates the costs of each DRG based on nationally representative samples. Therefore, the cost estimates from these countries facilitate reliable comparison of cost patterns with those from the UK. All costs are reported in euros and were adjusted for price differentials using the purchasing power parity method.^26^ Supplementary material provides more details on the costing methods (Supplementary material online, *Appendix pp 3–6*).

### Symptoms and cardiovascular disease events

The severity of symptoms (palpitations, syncope, shortness of breath, chest pain, general non-well-being, dizziness, fatigue, fear/anxiety, and others) was assessed using the European Heart Rhythm Association (EHRA) classification score, as recommended by the European Society of Cardiology (ESC) AF management guidelines.^10^ The EHRA classification system assigns patients to one of four classes based on the severity of their symptoms and the extent to which symptoms interfere with daily activities. The EHRA categories are EHRA I ‘no symptoms’, EHRA II ‘mild symptoms (normal daily activity not affected)’, EHRA III ‘severe symptoms (normal daily activity affected)’, and EHRA IV ‘disabling symptoms (normal daily activity discontinued)’. The treating physician was responsible for assigning the patient’s scores. We combined EHRA III, ‘severe symptoms’, and EHRA IV ‘disabling symptoms’ to a single class due to the relatively infrequent reporting of class four (EHRA IV).

Cardiovascular disease events included angina or non-ST-elevation myocardial infarction (NSTEMI), ST-segment elevation myocardial infarction (STEMI), new-onset or worsening of heart failure, thromboembolic events [stroke, transient ischaemic attach (TIA), peripheral embolism, pulmonary embolism, deep venous thrombosis, and other], and bleeding events (intracranial haemorrhage, major extracranial bleeding, and clinically relevant non-major bleeding).

### Statistical analysis

We present continuous variables as means with standard deviations and categorical variables as counts and proportions. Associations of symptoms and CVD events with changes in HRQOL and annual healthcare costs were estimated using fixed effects (FE) models with clustered standard errors. The annual changes in the number of ER admissions, cardiology visits, and internal medicine/GP visits were estimated using FE Poisson models, with clustered standard errors. The FE models use the longitudinal nature of the data and each study participant serves as their own control. An advantage of this type of model is that it simultaneously controls for both observable (time-variant and time-invariant) and unobservable (time-invariant) variables associated with each study participant, greatly reducing the risk of bias from omitted variables. All models were adjusted for the following time-varying covariates: type of AF (permanent, first diagnosed, paroxysmal, persistent, and long-standing persistent), age at each visit, medications used prior to the clinical visit, and calendar year to identify time-varying trends in the outcomes not captured by other variables in the model. We estimated changes in HRQOL between three time points: baseline, Follow-up 1, and Follow-up 2. For healthcare costs and healthcare visits, only changes between the first and second follow-up intervals were estimated. To examine differences in HRQOL, annual healthcare costs, and number of healthcare visits by sex and region, we included interaction terms between each type of CVD event and symptom with sex and separately with study regions. For all analyses, we excluded participants who died during the follow-up period but conducted sensitivity analyses to evaluate the impact of this exclusion on our results. When HRQOL was the outcome, our sensitivity analysis included all participants, allowing those who died to contribute to the estimates up to the point of their death. For healthcare costs as the outcome, we similarly included all participants and accounted for the costs associated with causes of death. Additionally, in separate sensitivity analyses across all outcomes, we used inverse probability weighting (IPW) methods.^27^ This approach assigned higher weights to participants similar to those who died during follow-up, to compensate for the absence of these participants (i.e. those who died) from the analysis. A difference was deemed statistically significant if *P* < 0.05. Additional details on the statistical analyses are provided in Supplementary material online, *Appendix (p 9)*.

### Missing data

We determined the percentage of missing data for each variable used in this study (see Supplementary material online, *Appendix p 8*). We used multiple imputation by chained equations to impute missing data. Predictive mean matching with 10 nearest neighbours was used to create 50 imputed data sets. Estimates derived from each imputed data set were combined using Rubin’s Rule.^28^ We present data using the imputed data set; the results of the complete case analyses are available in Supplementary material online, *Appendix (pp 10–16)*.

## Results

Between October 2013 and September 2016, a total of 10 249 AF patients were enrolled, (with 74% enrolled in 2015 and 2016), and were followed for 2 years. Among those, 971 patients died during follow-up. These 971 patients had lower HRQOL at the baseline visit than patients alive throughout the study, but total healthcare costs between the two groups were similar at the baseline visit (see Supplementary material online, *Appendix p 11*).

### Patient characteristics at baseline

*Table 1* presents the characteristics of the patients at the baseline visit. The mean age was 70 years, 4133 (40%) were female, and 5059 (49%) were enrolled from outpatient centres. Using the EHRA symptom classification score, 3653 (36%) had mild symptoms, and 1977 (19%) had severe or disabling symptoms. In terms of prior CVD events, 167 (2%) reported a history of angina/NSTEMI, 43 (<1%) STEMI, 84 (1%) new-onset/worsening heart failure, 32 (<1%) thromboembolic events, and 85 (1%) bleeding events. In regards to AF classification, 1575 (15%) had a first diagnosed AF, whereas 3404 (33%) were classified as permanent. The HAS-BLED score was 3 or higher in 1786 (17%) patients. The most common co-morbidities were hypertension 6258 (61%), valvular disease 5054 (49%), lipid disorders 4081 (40%), heart failure 3960 (39%), coronary artery disease 2775 (27%), and diabetes 2351 (23%). Of the behavioural risk factors, 877 (9%) were current smokers, 3220 (31%) were current alcohol drinkers, and 3698 (36%) exercised regularly. In terms of medications, 4462 (44%) were on a vitamin K antagonist, 2525 (25%) were on a non-vitamin K antagonist, and 2278 (22%) were taking an antiplatelet agent.

**Table 1:** Characteristics at the baseline visit, *n* = 10 249

Age, mean (SD)	70 (11.4)
Female sex, no (%)	4133 (40%)
Missing	0 (0%)
**Site of patient inclusion, no (%)**	
Outpatient/office based	5059 (49%)
Hospitalized	5190 (51%)
Missing	0 (0%)
**EHRA classification score, no (%)**	
No symptoms	4618 (45%)
Mild symptoms	3653 (36%)
Severe/disabling symptoms	1977 (19%)
Missing	0 (0%)
**Prior cardiovascular disease events, no (%)**	
Angina/NSTEMI	167 (2%)
Missing	0 (0%)
STEMI	43 (<1%)
Missing	0 (0%)
New-onset/worsening heart failure	84 (1%)
Missing	8 (<1%)
Thromboembolic events	32 (<1%)
Missing	3 (<1%)
Bleeding events	85 (1%)
Missing	3 (<1%)
**Clinical type of atrial fibrillation, no (%)**	
First diagnosed	1575 (15%)
Paroxysmal	2644 (26%)
Persistent	1996 (19%)
Long-standing persistent	453 (4%)
Permanent	3404 (33%)
Missing	177 (2%)
**HAS-BLED score, no (%)**	
Low bleeding risk (score of ≤2)	8463 (83%)
High bleeding risk (score of ≥3)	1786 (17%)
Missing	0 (0%)
**Clinical history, no (%)**	
Hypertension	6258 (61%)
Missing	84 (1%)
Coronary artery disease	2775 (27%)
Missing	579 (6%)
Myocardial infarction	1226 (12%)
Missing	579 (6%)
Heart failure	3960 (39%)
Missing	84 (1%)
Valvular disease	5054 (49%)
Missing	201 (2%)
Dilated cardiomyopathy	873 (9%)
Missing	116 (1%)
Hypertrophic cardiomyopathy	315 (3%)
Missing	122 (1%)
Congenital heart disease	107 (1%)
Missing	100 (1%)
Pulmonary arterial hypertension	691 (7%)
Missing	170 (2%)
Diabetes	2351 (23%)
Missing	65 (1%)
Lipid disorder	4081 (40%)
Missing	446 (4%)
Ischaemic stroke	626 (6%)
Missing	95 (1%)
Transient ischaemic attack	325 (3%)
Missing	95 (1%)
Peripheral vascular disease	792 (8%)
Missing	212 (2%)
Chronic kidney disease	1255 (12%)
Missing	75 (1%)
Chronic obstructive pulmonary disease	905 (9%)
Missing	80 (1%)
Malignancy	790 (8%)
Missing	70 (1%)
**Behavioural risk factors, no (%)**	
Current smoker	877 (9%)
Missing	742 (7%)
Current alcohol drinker	3220 (31%)
Missing	1192 (12%)
No regular exercise	3698 (36%)
Missing	1439 (14%)
**Medications received, no (%)**	
Vitamin K antagonists	4462 (44%)
Missing	77 (1%)
Non-vitamin K antagonist oral anticoagulants	2525 (25%)
Missing	47 (<1%)
Antiplatelet agent use	2278 (22%)
Missing	49 (<1%)

EHRA, European Heart Rhythm Association; NSTEMI, non-ST-elevation myocardial infarction; STEMI, ST-segment elevation myocardial infarction.

### Association of symptoms and cardiovascular disease events on health-related quality of life

*Table 2* presents the marginal effects [95% confidence interval (CI)] of symptoms and CVD events on changes in HRQOL. Compared with patients without symptoms, mild symptoms were associated with an EQ-5D utility decrement of 0.037 (95% CI −0.048, −0.026), *P* < 0.001, and severe/disabling symptoms with a decrement of 0.090 (95% CI −0.108, −0.072), *P* < 0.001.

**Table 2:** Marginal effects (95% CI) of symptoms and CVD events on HRQOL

	Health-related quality of life	
	Marginal effects (95% CI)	*P*-value
**Symptoms** ^a^		
No symptoms	Reference	
Mild symptoms	−0.037 (−0.048, −0.026)	*P* < 0.001
Severe/disabling symptoms	−0.090 (−0.108, −0.072)	*P* < 0.001
**Cardiovascular disease events**		
No cardiovascular disease events	Reference	
Angina or NSTEMI	−0.037 (−0.071, −0.003)	0.034
STEMI	−0.075 (−0.144, −0.006)	0.034
New-onset/worsening heart failure	−0.064 (−0.088, −0.039)	*P* < 0.001
Thromboembolic events	−0.071 (−0.115, −0.027)	0.002
Bleeding events	−0.031 (−0.059, −0.003)	0.029

Fixed effects models adjusted for unknown time-invariant confounders and for the following observed time-variant confounders: clinical type of atrial fibrillation, age, medications used before each visit, and time period.

CI, confidence interval; NSTEMI, non-ST-elevation myocardial infarction; STEMI, ST-segment elevation myocardial infarction.

^a^Symptoms measured using European Heart Rhythm Association (EHRA) classification score.

All CVD events were associated with decrements in HRQOL. Angina or NSTEMI was associated with EQ-5D utility decrement of 0.037 (95% CI −0.071, −0.003), *P* = 0.034; STEMI was associated with a decrement of 0.075 (95% CI −0.144, −0.006), *P* = 0.034; new-onset worsening heart failure was associated with a decrement of 0.064 (95% CI −0.088, −0.039), *P* < 0.001; thromboembolic events was associated with a decrement of 0.071 (95% CI −0.115, −0.027), *P* = 0.002, and bleeding events with a decrement of 0.031 (95% CI −0.059, 0.003), *P* = 0.029.

### Association of symptoms and cardiovascular disease events on healthcare visits

*Table 3* presents incident rate ratios (IRRs) and 95% CI of symptoms and CVD events on rates of ER admissions, cardiology visits, and internal medicine/GP visits. European Heart Rhythm Association symptom status was significantly associated with the rates of healthcare visits, especially AF patients with severe/disabling symptoms. Compared with patients without symptoms, rates of ER admissions for patients with severe/disabling symptoms were 2.14 times greater [(95% CI 1.79, 2.56), *P* < 0.001], rates of cardiology visits 1.23 times greater [(95% CI 1.12, 1.34), *P* < 0.001], and internal medicine/GP visits 1.20 times greater [(95% CI 1.05, 1.38), *P* = 0.007].

**Table 3:** Incidence rate ratios (95% CI) of number of ER admissions, cardiology visits, and internal medicine/GP visits

	No. of ER admissions		No. of cardiology visits		No. of internal medicine/GP visits	
	IRR (95% CI)	*P*-value	IRR (95% CI)	*P*-value	IRR (95% CI)	*P*-value
**Symptoms^a^**						
No symptoms	Reference		Reference		Reference	
Mild symptoms	1.51 (1.30, 1.77)	*P* < 0.001	1.13 (1.06, 1.20)	*P* < 0.001	1.19 (1.09, 1.29)	*P* < 0.001
Severe/disabling symptoms	2.14 (1.79, 2.56)	*P* < 0.001	1.23 (1.12, 1.34)	*P* < 0.001	1.20 (1.05,1.38)	0.007
**Cardiovascular disease events**						
No cardiovascular disease events	Reference		Reference		Reference	
Angina or NSTEMI	2.49 (1.78, 3.49)	*P* < 0.001	1.05 (0.88, 1.25)	0.568	0.92 (0.70, 1.21)	0.554
STEMI	1.72 (0.78, 3.77)	0.176	1.03 (0.66, 1.61)	0.894	1.02 (0.33, 3.14)	0.978
New-onset/worsening heart Failure	1.71 (1.38, 2.11)	*P* < 0.001	1.32 (1.19, 1.47)	*P* < 0.001	1.11 (0.94, 1.31)	0.218
Thromboembolic events	2.05 (1.38, 3.04)	*P* < 0.001	1.21 (1.01, 1.46)	0.044	1.44 (1.11, 1.88)	0.007
Bleeding events	2.48 (1.82, 3.37)	*P* < 0.001	1.13 (0.99, 1.29)	0.079	1.33 (1.07, 1.65)	0.011

Fixed effects models adjusted for unknown time-invariant confounders and for the following observed time-variant confounders: clinical type of atrial fibrillation, age, medications used before each visit, and time period.

CI, confidence interval; GP, general practitioner; ER, emergency room; IRR, incident rate ratio; NSTEMI, non-ST-elevation myocardial infarction; STEMI, ST-segment elevation myocardial infarction.

^a^Symptoms measured using European Heart Rhythm Association (EHRA) classification score.

Atrial fibrillation patients with angina or NSTEMI had the highest rate of ER admissions, 2.49 times greater [(95% CI 1.78, 3.49), *P* < 0.001] than patients without angina or NSTEMI. This was followed by AF patients with bleeding events [IRR: 2.48 (95% CI 1.82, 3.37), *P* < 0.001]. The rate of ER admissions for AF patients with thromboembolic events was 2.05 times greater [(95% CI 1.38, 3.04), *P* < 0.001], and admissions for new-onset/worsening heart failure were 1.71 times greater [(95% CI 1.38, 2.11), *P* < 0.001]. By contrast, the rate of ER admissions did not change significantly for AF patients with STEMI compared with those without STEMI.

The rate of cardiology visits was not significantly different for CVD events, except in cases of new-onset or worsening heart failure. Similarly, except for thromboembolic and bleeding events, the rate of internal medicine/GP visits was not significantly different for AF patients with CVD events compared with those without.

### Association of symptoms and cardiovascular disease events on healthcare costs

Atrial fibrillation patients experiencing severe/disabling symptoms incurred an increased annual cost of €544 (95% CI €225, €862), *P* = 0.001; *Table 4*. By contrast, healthcare costs for patients with mild symptoms were not significantly different from patients without symptoms.

**Table 4:** Marginal effects (95% CI) of symptoms and CVD events on healthcare costs (€)

	Healthcare costs (€)	
	Marginal effects (95% CI)	*P*-value
**Symptoms** ^a^		
No symptoms	Reference	
Mild symptoms	157 (−24, 339)	0.090
Severe/disabling symptoms	544 (225, 862)	0.001
**Cardiovascular disease events**		
No cardiovascular disease events	Reference	
Angina or NSTEMI	5823 (4757, 6889)	*P* < 0.001
STEMI	11 718 (8497, 14 939)	*P* < 0.001
New-onset/worsening heart failure	3689 (3219, 4158)	*P* < 0.001
Thromboembolic events	3182 (2483, 3881)	*P* < 0.001
Bleeding events	3792 (3315, 4270)	*P* < 0.001

Fixed effects models adjusted for unknown time-invariant confounders and for the following observed time-variant confounders: clinical type of atrial fibrillation, age, medications used before each visit, and time period.

CI, confidence interval; NSTEMI, non-ST-elevation myocardial infarction; STEMI, ST-segment elevation myocardial infarction.

^a^Symptoms measured using European Heart Rhythm Association (EHRA) classification score.

All CVD events were associated with significant increases in healthcare costs. Compared with patients without CVD events, AF patients with angina/NSTEMI incurred an increased annual cost of €5823 (95% CI €4757, €6889), *P* < 0.001. Patients experiencing STEMI incurred notably higher healthcare costs: an increase of €11 718 (95% CI €8497, €14 939), *P* < 0.001. Atrial fibrillation patients with bleeding events or new-onset/worsening heart failure had similar increases in their annual healthcare costs: €3792 (95% CI €3315, €4270), *P* < 0.001 and €3689 (95% CI €3219, €4158), *P* < 0.001, respectively, whereas patients with thromboembolic events incurred an annual increase of €3182 (95% CI €2483, €3881), *P* < 0.001. Healthcare costs were primarily driven by inpatient costs (see Supplementary material online, *Appendix p 12*).

### Variations by region and biological sex

**Figure 1:**
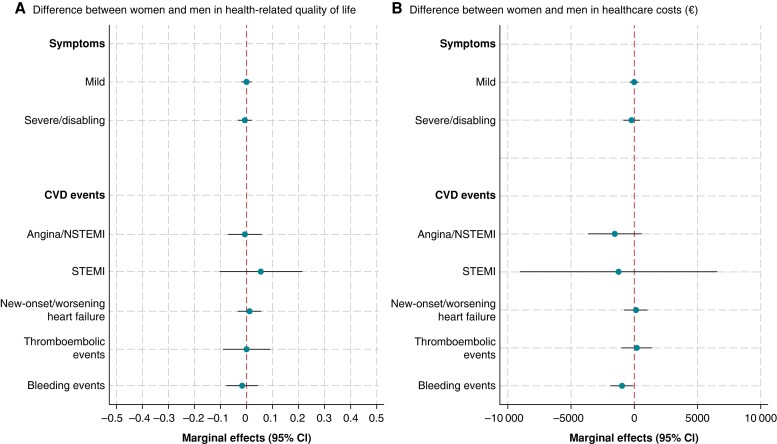
Marginal effects (95% CI) of symptoms and CVD events on HRQOL and healthcare costs (€) of women relative to men. Symptoms measured using EHRA classification score. FE models with interaction terms for symptoms and CVD events with biological sex. Models adjusted for unknown time-invariant confounders and for the following observed time-variant confounders: clinical type of AF, age, medications used before each visit, and time period. Men are the reference category. AF, atrial fibrillation; CI, confidence interval; CVD, cardiovascular disease; EHRA, European Heart Rhythm Association; FE, fixed effects; HRQOL, health-related quality of life; NSTEMI, non-ST-elevation myocardial infarction; STEMI, ST-segment elevation myocardial infarction.

**Figure 2:**
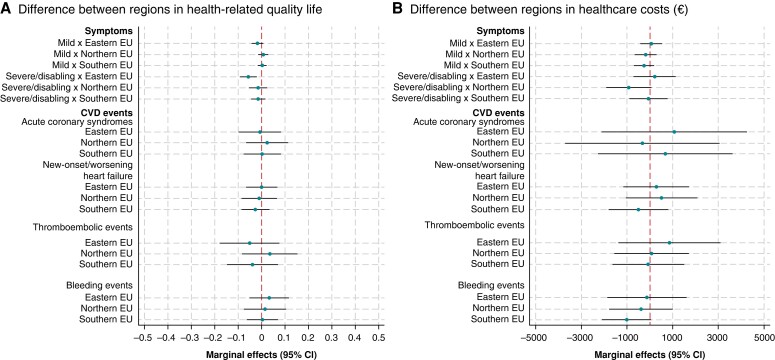
Marginal effects (95% CI) of symptoms and CVD events on HRQOL and healthcare costs (€) by region. Acute coronary syndromes includes Angina/NSTEMI and STEMI (STEMI was not estimated in Northern EU due to few events). Symptoms measured using EHRA classification score. FE models with interaction terms symptoms and CVD events with region. Models adjusted for unknown time-invariant confounders and for the following observed time-variant confounders: clinical type of AF, age, medications used before each visit, and time period. Western EU is the reference category. Northern EU countries: Denmark, Estonia, Latvia, Norway, and the UK; Western EU countries: Belgium, France, Germany, the Netherlands, and Switzerland; Eastern EU countries: Bulgaria, Czech Republic, Georgia, Kazakhstan, Kyrgyzstan, Poland, Romania, and Russia; Southern EU countries: Albania, North Macedonia, Italy, Malta, Montenegro, Portugal, Serbia, Spain, and Turkey. AF, atrial fibrillation; CI, confidence interval; CVD, cardiovascular disease; EHRA, European Heart Rhythm Association; FE, fixed effects; HRQOL, health-related quality of life; NSTEMI, non-ST-elevation myocardial infarction; STEMI, ST-segment elevation myocardial infarction.

We did not observe sex differences in HRQOL or healthcare costs (*Figure 1*) or significant variations in these outcomes by regions within Europe (*Figure 2*). Similarly, we did not observe significant differences in the number of ER admissions, cardiology visits, and internal medicine/GP visits by sex or region (see Supplementary material online, *Appendix pp 20–21*).

### Sensitivity analyses

Using tariffs from Spain, Germany, and Slovenia showed similar trends in the decline of HRQOL to those in the UK (see Supplementary material online, *Appendix p 14*). For example, severe/disabling symptoms led to larger utility decrements than mild symptoms. Cardiovascular disease events such as STEMI, new-onset/worsening heart failure, and thromboembolic events led to highly significant negative decrements.

Using costs from Spain, Germany, and Slovenia did not alter any of our conclusions (see Supplementary material online, *Appendix p 16*). In all three countries, the patterns of healthcare cost increases due to symptoms and CVD were broadly similar to those in the UK. For example, CVD events were associated with significant cost increases in all four countries, and patients experiencing severe/disabling symptoms incurred higher annual cost increases compared with patients experiencing mild symptoms. These results suggest that severe/disabling AF symptoms and CVD events are associated with significant increases in resource utilization

Including all participants in the FE regressions yielded marginal effects on HRQOL and healthcare costs that were similar to those from models excluding deceased participants (see Supplementary material online, *Appendix pp 15 and 17*). Similarly, using IPW methods resulted in estimates that were comparable with those from unweighted analyses (see Supplementary material online, *Appendix pp 18 and 19*).

## Discussion

Our study has three major findings. First, symptoms and CVD events were associated with decrements in HRQOL, with STEMI, thromboembolic events, new-onset/worsening heart failure, and severe/disabling symptoms associated with significant decrements. Second, CVD events and, to a lesser extent, symptoms were found to be associated with a significant increase in healthcare costs. Notably, the costs due to inpatient care were the primary driver of cost increases. Third, we did not observe significant differences in HRQOL or overall healthcare costs between European regions or by sex.

Previous studies have reported negative associations between symptom severity measured using the EHRA symptom score and HRQOL, but these studies were all cross-sectional. For instance, severe/disabling EHRA symptoms were associated with a 0.069 reduction in EQ-5D [(95% CI −0.118, −0.019); *P* = 0.006] in the Swiss-AF study.^29^ In a sub-sample of 2000 US patients from the Outcomes Registry for Better Informed Treatment of Atrial Fibrillation registry, higher EHRA symptom scores were correlated with lower patient-reported HRQOL measured using the Atrial Fibrillation Effect on Quality-of-Life score (i.e. Spearman correlation coefficient of −0.39).^30^ A negative correlation between EHRA symptom score and EQ-5D was reported in 362 AF patients from the UK.^31^ Moderate correlations with the components of EQ-5D were reported using data on 6196 European patients from the Prevention of Thromboembolic Events-European Registry.^32^ By contrast, in 1218 new-onset AF patients from the ARENA (Atrial Fibrillation RhineNeckar Region) trial, symptom severity was not associated with differences in HRQOL.^33^

Fewer studies have examined the relationships between CVD events and HRQOL in AF patients. In the ARENA trial, history of coronary artery disease was associated with 0.056 reduction in HRQOL [(95% CI −0.089, −0.023), *P* = 0.001], whereas a history of stroke/TIA had modest non-significant associations [estimate of −0.039 (95% CI −0.081, 0.003); *P* = 0.068].^33^ By contrast, data from the Euro heart survey reported stroke as having the largest decrement in HRQOL [−0.272 (95% CI −0.345, −0.198)].^34^ However, both the ARENA trial and the Euro Heart Survey analyses were cross-sectional and the latter study enrolled participants between 2003 and 2004, prior to the introduction of novel oral anticoagulation therapies. These differences between studies may explain the difference in findings.

We found that CVD events were costly, ranging from an annual cost of €3182 for AF patients with thromboembolic events to €11 718 for AF patients with STEMI. A study of AF patients from Sweden and Germany also reported that coronary artery disease, cerebrovascular disease, and heart failure were significant contributors to patients’ healthcare costs; in their cross-sectional analysis, the authors reported relative cost increases ranging from 50% (coronary artery disease and heart failure) to 80% (cerebrovascular disease).^35^ A Danish cost-of-illness study that separately calculated the cost of ischaemic stroke reported an average total cost of €49 576 (in 2013 euros) over 3 years of follow-up for ischaemic stroke patients with AF (after removing homecare costs and productivity losses).^36^ The average hospital costs for ischaemic stroke patients with a history of AF from the Oxford Vascular Study were £12042 (in 2008/2009 UK pounds sterling).^37^ Consistent with previous costing analyses of AF, we found that the increased healthcare costs from concomitant CVDs were primarily driven by inpatient costs.^36,38–42^ Moreover, we observed that patients with symptoms and CVD events had significantly more ER admissions over the course of a year than AF patients without such symptoms and events. These findings are consistent with a recent study of AF patients from the UK, whereby heart failure, ischaemic heart disease, and stroke were among the most common reasons for hospitalization following a diagnosis of AF.^43^

We did not observe differences in the association between symptoms or CVD events and HRQOL or healthcare costs by sex or European regions. Using administrative healthcare data from Scotland, Ciminata *et al*.^41^ also found no differences between women and men in inpatient, outpatient, and prescription costs. However, in their analysis of healthcare costs associated with AF, care home costs were higher in women (5% of total healthcare costs in males vs. 7% of total costs among women).^41^ The authors attributed this difference to a higher likelihood of elderly women residing in care homes. We did not include care home costs in our analysis. Moreover, while studies have reported that women with AF report poorer quality of life,^44,45^ to our knowledge, studies have not examined the association of symptoms or CVD events with HRQOL or healthcare costs in women compared with men with AF. Therefore, our results will require independent replication. The Euro Heart Survey^6^ found that the predictors of healthcare costs varied across the five EU countries examined; however, it is difficult to make meaningful comparisons because the sample sizes were small in some countries, treatment patterns have changed, and cross-sectional analyses are more vulnerable to bias.

### Limitations

Our study has some limitations. The first concerns the generalizability of our study population. The patients in our study were under the care of a cardiologist and therefore may not be representative of AF patients under the care of GPs. Atrial fibrillation patients who experience minor symptoms or no symptoms are unlikely to be included in our study sample since these patients often do not seek medical attention. Moreover, the enrolment of patients for this study was completed before the dissemination of the 2020 ESC guidelines for managing AF patients,^10^ which emphasized the importance of systematic screening and a comprehensive characterization of AF patients using the 4S-AF scheme, rather than relying on a single domain classification of AF. The 2020 guidelines also recommend a shift towards a more streamlined, integrated, and multidisciplinary model of care, following the ‘Atrial Fibrillation Better Care’ pathway.^46^ As a consequence, there may have been changes in clinical practice since the initiation of this study, potentially affecting the generalizability of our findings. However, the magnitude and impact of such changes on the relevance of our results remain uncertain. We excluded patients who died during the 2-year follow-up because we wanted to examine temporal changes in HRQOL and healthcare costs. However, sensitivity analyses that included these patients produced comparable estimates. We used unit costs from the UK with sensitivity analysis using costs from Spain, Germany, and Slovenia. A drawback of this approach is that the cost estimates may not reflect ‘actual’ care costs in each country. Nonetheless, our objective was to examine the associations of AF symptoms and CVD events on resource use intensity for a cross-EU population. Applying country-specific prices to each participant would complicate the interpretation of cost differences, because it would be challenging to distinguish between cost differences due to varying prices across countries (often influenced by country-specific differences in care valuation, wages, and data quality) vs. intensity of resource use. Furthermore, the limited number of patients within each country precluded us from conducting robust country-specific analyses. Additionally, the absence of data on the duration of hospital stays during the follow-up likely led to an underestimation of the intensity of resource use. We did not include the costs of long-term residential or nursing home care as this information was not captured in the registry. A previous study of stroke patients with a history of AF reported that the average annual costs of institutionalization post-stroke was £6880 (in 2008/2009 UK pounds sterling).^42^ While the percent of missing data was low for most variables, the percent missing was quite high for HRQOL, especially at 24 months. However, our results from the imputed data sets were not appreciably different from the complete case analyses. We used a generic instrument of HRQOL, which has the disadvantage that it could be less sensitive to the effects of AF compared with instruments developed specifically for AF. However, a systematic review of the measurement properties of AF-specific instruments was unable to recommend an instrument due to the low ratings across dimensions they examined.^47^ Moreover, generic instruments such as EQ-5D-5L allow for comparisons of HRQOL in patients with different diseases, have been extensively validated, and are recommended for cost-effectiveness analyses.

To our knowledge, this is the first study to examine changes over time by using repeated measurements of HRQOL, healthcare resource use, and patient characteristics in over 9000 AF patients. Previous studies primarily used cross-sectional designs, while the EORP-AF General Long-Term Registry has 2 years of follow-up, allowing us to examine changes in HRQOL and costs over time. Moreover, the FE models account for unobserved time-invariant differences between patients, greatly reducing the risk of confounding that is present in analyses that do not take into account the repeat measurements.

In conclusion, we find that symptoms and CVD events are associated with a high burden on both AF patients and healthcare systems throughout Europe.

## Supplementary Material

euae146_Supplementary_Data

## Data Availability

The data underlying this article were provided by the European Society of Cardiology’s Atrial Fibrillation General Registry by permission. Data will be shared on request to the corresponding author with permission of the European Society of Cardiology’s Atrial Fibrillation General Registry.
