# Supplementary material for: Health-related quality of life and healthcare costs of symptoms and cardiovascular disease events in patients with atrial fibrillation: a longitudinal analysis of 27 countries from the EURObservational Research Programme on Atrial Fibrillation general long-term registry
Source: Europace. 2024 May 29;26(6):euae146. doi: 10.1093/europace/euae146 (PMC11164571; doi:10.1093/europace/euae146)
Supplement: euae146_Supplementary_Data [file euae146_supplementary_data.pdf]

Supplemental appendix

Table of Contents

Table A1 countries included in the EORP-AF Long-Term General Registry 2

Supplemental methods 3

Deriving healthcare costs 3

Table A2: Healthcare costs 3

Table A3 List of medication net ingredient costs (NIC) per item prescribed 6

Missing data 7

Table A4 Response level data for EQ-5D-5L and mean EQ-5D-VAS at each follow-up time 7

Table A5 Percent missing at each time point, N=10249 8

Statistical analysis 9

Supplemental Results 10

Table A6 Mean (SD) EQ-5D-5L, total healthcare costs, and number of healthcare visits by time 10

Table A7 Mean (standard deviation) of health-related quality of life and total healthcare costs in patients that died during follow-up compared to patients that were alive throughout the study 11

Table A8 Marginal effects (95% CI) of symptoms and cardiovascular disease events on total healthcare costs, inpatient costs, outpatient costs, and medication costs in imputed vs complete case analysis 12

Table A9 Marginal effects (95% CI) of symptoms and cardiovascular disease events on health-related quality of life and healthcare costs (€) in imputed vs complete case analysis 13

Sensitivity analyses 14

Table A10 Marginal effects (95% CI) of symptoms and cardiovascular disease events on health-related quality of life using tariffs from the United Kingdom, Spain, Germany, and Slovenia 14

Table A11 Marginal effects (95% CI) of symptoms and cardiovascular disease events on health-related quality of life comparing estimates when all participants included vs deceased removed 15

Table A12 Marginal effects (95% CI) of symptoms and cardiovascular disease events on healthcare costs (€) comparing costs from the United Kingdom, Spain, Germany, and Slovenia 16

Table A13 Marginal effects (95% CI) of symptoms and cardiovascular disease events on healthcare costs (€)) when deceased participants excluded vs included 17

Table A14 Marginal effects (95% CI) of symptoms and cardiovascular disease events on health-related quality of life and healthcare costs (€)) comparing unweighted to IPW estimates 18

Table A15 Incidence rate ratios (95% CI) of symptoms and cardiovascular disease events on healthcare visits comparing unweighted to IPW estimates 19

Table A16 Incidence rate ratios (95% CI) of number of ER admissions, cardiology visits, internal medicine/GP visits by region 20

Table A17 Incidence rate ratios (95% CI) of number of ER admissions, cardiology visits, internal medicine/GP visits by sex 21

STROBE Statement—Checklist of items that should be included in reports of *cohort studies* 22

References 25

Table A1 countries included in the EORP-AF Long-Term General Registry

Northern Europe	Western Europe	Eastern Europe	Southern Europe
Denmark<br>Estonia<br>Latvia<br>Norway<br>United Kingdom	Belgium<br>France<br>Germany<br>Netherlands<br>Switzerland	Bulgaria<br>Czech Republic<br>Georgia<br>Kazakhstan<br>Kyrgyzstan<br>Poland<br>Romania<br>Russia	Albania<br>Italy<br>Malta<br>Montenegro<br>North Macedonia<br>Portugal<br>Serbia<br>Spain<br>Turkey

Notes: Countries were classified into European regions according to the United Nations geoscheme

Supplemental methods

Deriving healthcare costs

Healthcare use consisted of inpatient admissions, outpatient visits, and medications. All resource utilisation in the registry during follow-up were costed. To combine the various types of resource use into a single metric and enable cross-country comparisons of resource volume, we valued all resource use in the same currency, UK NHS costs for 2018/19.[1,2] Inpatient resource use were assigned a Healthcare Resource Group (HRG) using the 2018/2019 reference cost grouper.[1] HRGs are UK groups of diagnoses (based on International Classification of Diseases-10) and procedure (OPCS) codes that group clinically similar treatments that use similar levels of healthcare resources. Inpatient care costs were then calculated using the 2018/2019 values using the NHS reference costs.[3] Table A2 lists the HRGs and costs used in our analyses. Medications taken prior to each visits were costed using prices from the Prescription Cost Analysis: England 2018.[2] Table A3 lists the medications and their prices. Costs were converted into euros using the purchasing power parity (PPP) method, with EU-27 as the reference.

Table A2: Healthcare costs

HRG	HRG description	Cost (£)	Cost (€)
EB07	Arrhythmia or Conduction Disorders, with CC Score 0-3	957	944
EY42	Complex Cardiac Catheterisation with CC Score 0-1	3038	2995
EB07	Arrhythmia or Conduction Disorders, with CC Score 0-3	957	944
EB03	Heart Failure or Shock, with CC Score 0-3	1962	1934
EY31	Standard Percutaneous Transluminal Ablation of Heart with CC Score 0-2	3459	3411
AA35	Stroke with CC Score 0-3	3338	3291
EB10	Actual or Suspected Myocardial Infarction, with CC Score 0-3	1479	1458
EY41	Standard Percutaneous Transluminal Coronary Angioplasty with CC Score 0-3	2881	2841
EY08	Implantation of Single-Chamber Pacemaker with CC Score 0-2	3532	3482
EY30	Complex Percutaneous Transluminal Ablation of Heart with CC Score 0-2	4322	4261
EY51	Electrocardiogram Monitoring or Stress Testing	829	817
EY32	Percutaneous Diagnostic Electrophysiology Studies with CC Score 0-1	3035	2992
EY22	Complex Other Percutaneous Transluminal Repair of Acquired Defect of Heart with CC Score 0-4	6507	6416
ED25	Standard, Single Heart Valve Replacement or Repair, with CC Score 0-5	11808	11642
EB14	Other Acquired Cardiac Conditions with CC Score 0-2	1813	1788
EY02	Implantation of Cardioverter Defibrillator with CC Score 0-8	4983	4913
EB04	Hypertension	614	605
EB13	Angina with CC Score 0-3	720	710
DZ11	Lobar, Atypical or Viral Pneumonia, without Interventions, with CC Score 0-3	1777	1752
DZ19	Other Respiratory Disorders without Interventions, with CC Score 0-4	629	620
EY07	Implantation of Single-Chamber Pacemaker with Other Percutaneous Intervention, with CC Score 0-5	6910	6813
EB08	Syncope or Collapse, with CC Score 0-3	883	871
ED28	Standard Coronary Artery Bypass Graft with CC Score 0-4	10352	10207
DZ65	Chronic Obstructive Pulmonary Disease or Bronchitis, without Interventions, with CC Score 0-4	1187	1170
EY01	Implantation of Cardioverter Defibrillator with Cardiac Resynchronisation Therapy, with CC Score 0-8	4765	4698
EB12	Unspecified Chest Pain with CC Score 0-4	408	402
ED24	Complex, Single Heart Valve Replacement or Repair, with CC Score 0-5	14693	14487
ED23	Standard, Coronary Artery Bypass Graft with Single Heart Valve Replacement or Repair, with CC Score 0-5	14011	13815
ED21	Standard, Repair or Replacement, of Multiple Heart Valves, with CC Score 0-7	15253	15039
DZ11	Lobar, Atypical or Viral Pneumonia, with Single Intervention, with CC Score 0-7	1777	1752
LA04	Kidney or Urinary Tract Infections, without Interventions, with CC Score 0-1	1614	1591
ED27	Major Coronary Artery Bypass Graft with CC Score 0-4	11360	11201
EB02	Endocarditis with CC Score 0-4	4399	4337
ED31	Standard, Other Operations on Heart or Pericardium, with CC Score 0-4	5033	4962
WJ06	Sepsis without Interventions, with CC Score 0-4	2213	2182
ED20	Complex, Repair or Replacement, of Multiple Heart Valves, with CC Score 0-7	18490	18231
KB02	Diabetes with Hyperglycaemic Disorders, with CC Score 0-1	1067	1052

ED05	Standard Heart Transplant	57348	56544
DZ11	Lobar, Atypical or Viral Pneumonia, with Multiple Interventions, with CC Score 0-8	1777	1752
DZ20	Pulmonary Oedema without Interventions, with CC Score 0-5	1476	1455
AA26	Muscular, Balance, Cranial or Peripheral Nerve Disorders, Epilepsy or Head Injury, with CC Score 0-2	1398	1378
DZ65	Chronic Obstructive Pulmonary Disease or Bronchitis, with Single Intervention, with CC Score 0-4	1187	1170
EY03	Implantation of Biventricular Pacemaker with Other Percutaneous Intervention	10555	10407
EY50	Complex Echocardiogram	1423	1403
FD04	Nutritional Disorders without Interventions, with CC Score 0-1	2004	1976
FD10	Non-Malignant Gastrointestinal Tract Disorders without Interventions, with CC Score 0-2	1437	1417
HD24	Non-Inflammatory, Bone or Joint Disorders, with CC Score 0-1	2236	2205
JD07	Skin Disorders without Interventions, with CC Score 0-1	1548	1526
LA04	Kidney or Urinary Tract Infections, with Interventions, with CC Score 0-2	1614	1591
SA09	Other Red Blood Cell Disorders with CC Score 0-1	1170	1154
ED26	Complex Coronary Artery Bypass Graft with CC Score 0-4	13179	12994
EY04	Implantation of Biventricular Pacemaker with CC Score 0-5	4870	4802
DZ16	Pleural Effusion without Interventions, with CC Score 0-5	1798	1773
DZ65	Chronic Obstructive Pulmonary Disease or Bronchitis, with Multiple Interventions, with CC Score 0-8	1187	1170
ED22	Complex, Coronary Artery Bypass Graft with Single Heart Valve Replacement or Repair, with CC Score 0-5	16699	16465
ED30	Complex, Other Operations on Heart or Pericardium, with CC Score 0-4	9676	9540
FD04	Nutritional Disorders with Interventions, with CC Score 0-1	2004	1976
HD26	Musculoskeletal Signs or Symptoms, with CC Score 0-3	940	927
JA12	Malignant Breast Disorders without Interventions, with CC Score 0-1	2390	2356
WH17	Admission Related to Social Factors without Interventions, with CC Score 0	1083	1068
WJ07	Fever of Unknown Origin without Interventions, with CC Score 0-3	961	948
YQ50	Peripheral Vascular Disorders with CC Score 0-1	1861	1835
DZ19	Other Respiratory Disorders with Single Intervention, with CC Score 0-4	629	620
LA09	General Renal Disorders without Interventions, with CC Score 0-2	1341	1322
WJ06	Sepsis with Single Intervention, with CC Score 0-4	2213	2182
WJ07	Fever of Unknown Origin with Interventions, with CC Score 0-3	961	948
DZ20	Pulmonary Oedema with Interventions	1476	1455
EB06	Cardiac Valve Disorders with CC Score 0-4	2377	2344
FD05	Abdominal Pain without Interventions	641	632
WH13	Abnormal Findings without Diagnosis, without Interventions, with CC Score 0	703	693
CB02	Non-Malignant, Ear, Nose, Mouth, Throat or Neck Disorders, with Interventions, with CC Score 0	876	864
DZ65	Chronic Obstructive Pulmonary Disease or Bronchitis, with length of stay 1 day or less, Discharged Home	1187	1170
FD02	Inflammatory Bowel Disease with Multiple Interventions, with CC Score 0-2	1633	1610
FD05	Abdominal Pain with Interventions	641	632
KA07	Non-Surgical Thyroid Disorders with CC Score 0-1	1254	1236
LB38	Unspecified Haematuria with Interventions, with CC Score 0-2	1179	1162
LB38	Unspecified Haematuria without Interventions, with CC Score 0-3	1179	1162
WH08	Unspecified Pain with CC Score 0	1005	991
WH09	Tendency to Fall, Senility or Other Conditions Affecting Cognitive Functions, without Interventions, with CC Score 0-1	1479	1458
CB02	Non-Malignant, Ear, Nose, Mouth, Throat or Neck Disorders, without Interventions, with CC Score 0	876	864
DZ26	Pneumothorax or Intrathoracic Injuries, without Interventions, with CC Score 0-2	1678	1654
EB05	Cardiac Arrest with CC Score 0-4	1597	1575
FD02	Inflammatory Bowel Disease without Interventions, with CC Score 0	1633	1610
FD10	Non-Malignant Gastrointestinal Tract Disorders with Single Intervention, with CC Score 0-2	1437	1417
GC17	Non-Malignant, Hepatobiliary or Pancreatic Disorders, without Interventions, with CC Score 0-1	1743	1719
HD21	Soft Tissue Disorders with CC Score 0-2	648	639
HD23	Inflammatory, Spine, Joint or Connective Tissue Disorders, with CC Score 0-2	1341	1322
JD07	Skin Disorders with Interventions, with CC Score 0-3	1548	1526

LA07	Acute Kidney Injury without Interventions, with CC Score 0-3	1849	1823
WH53	Follow-up Examination for Other Conditions, without Interventions	688	678
YQ51	Deep Vein Thrombosis with CC Score 0-2	908	895
AA25	Cerebral Degenerations or Miscellaneous Disorders of Nervous System, with CC Score 0-4	1817	1792
AA29	Transient Ischaemic Attack with CC Score 0-4	965	951
AA31	Headache, Migraine or Cerebrospinal Fluid Leak, with CC Score 0-6	613	604
BZ24	Non-Surgical Ophthalmology with Interventions	1087	1072
DZ09	Pulmonary Embolus without Interventions, with CC Score 0-2	1415	1395
DZ16	Pleural Effusion with Single Intervention, with CC Score 0-5	1798	1773
DZ17	Respiratory Neoplasms with Multiple Interventions, with CC Score 0-5	2296	2264
DZ19	Other Respiratory Disorders with Multiple Interventions	629	620
DZ22	Unspecified Acute Lower Respiratory Infection without Interventions, with CC Score 0-4	1130	1114
DZ27	Respiratory Failure without Interventions, with CC Score 0-5	1986	1958
EC22	Electrocardiogram Monitoring or Stress Testing, for Congenital Heart Disease	1474	1453
FD03	Gastrointestinal Bleed without Interventions, with CC Score 0-4	1214	1197
FD10	Non-Malignant Gastrointestinal Tract Disorders with Multiple Interventions, with CC Score 0-2	1437	1417
GC12	Malignant, Hepatobiliary or Pancreatic Disorders, without Interventions, with CC Score 0	2287	2255
HC27	Degenerative Spinal Conditions without Interventions, with CC Score 0-2	1210	1193
HC31	Spinal Infection without Interventions, with CC Score 0-2	5115	5043
HC32	Low Back Pain with Interventions	830	818
HE11	Hip Fracture without Interventions, with CC Score 0-3	2995	2953
HE32	Other Injury of Foot without Interventions, with CC Score 0-1	1005	991
JA12	Malignant Breast Disorders with Interventions, with CC Score 0-2	2390	2356
KC04	Inborn Errors of Metabolism with CC Score 0-2	1552	1530
KC05	Fluid or Electrolyte Disorders, with Interventions, with CC Score 0-4	1319	1301
KC05	Fluid or Electrolyte Disorders, without Interventions, with CC Score 0-1	1319	1301
LA09	General Renal Disorders with Interventions, with CC Score 0-2	1341	1322
LB28	Non-Malignant Prostate Disorders without Interventions, with CC Score 0-2	1402	1382
MB09	Non-Malignant Gynaecological Disorders with Interventions, with CC Score 0-2	1225	1208
SA02	Coagulation Defect with CC Score 0-1	2404	2370
WD05	Neurotic, Stress-Related or Somatoform Disorders, treated by a Non-Specialist Mental Health Service Provider	934	921
WD08	Mental and Behavioural Disorders Due to Drug or Alcohol Use, treated by a Non-Specialist Mental Health Service Provider	1121	1105
WH10	Unspecified Oedema with CC Score 0-1	598	590
WH13	Abnormal Findings without Diagnosis, with Interventions	703	693
WH15	Special Screening, Examinations or Other Genetic Disorders	707	697
WH16	Observation or Counselling, with CC Score 0	962	949
WJ03	Standard Infectious Diseases with Single Intervention, with CC Score 0-3	1107	1091
WJ06	Sepsis with Multiple Interventions, with CC Score 0-4	2213	2182

Table A3 List of medication net ingredient costs (NIC) per item prescribed

ATC	Drug name	BNF chemical name	NIC (£)	NIC (€)
B01AA03	Warfarin Sod_Tab 1mg	Warfarin Sodium	1	1
B01AA04	Marcoumar_Tab 3mg	Phenprocoumon	75	74
B01AA07	Sinthrome_Tab 1mg	Acenocoumarol	5	5
B01AA02	Phenindione_Tab 25mg	Phenindione	1869	1843
B01AE07	Pradaxa_Cap 110mg	Dabigatran Etexilate	46	45
B01AF01	Xarelto_Tab 20mg	Rivaroxaban	52	51
B01AF02	Eliquis_Tab 5mg	Apixaban	52	51
B01AF03	Lixiana_Tab 60mg	Edoxaban	52	51
B01AB01	Heparin Sod_Inj 5,000u/ml 1ml Amp	Heparin Sodium	89	88
B01AB04	Fragmin_Inj 25,000u/ml 0.2ml Pfs	Dalteparin Sodium	53	52
B01AX05	Arixtra_Inj 7.5mg/0.6ml Pfs	Fondaparinux Sodium	230	227
B01AC06	Aspirin Disper_Tab 75mg	Aspirin	1	1
B01AC04	Clopidogrel_Tab 75mg	Clopidogrel	1	1
B01AC22	Efient_Tab 10mg	Prasugrel	52	51
B01AC24	Brilique_Tab 90mg	Ticagrelor	54	53
B01AC05	Ticlopidine HCl_Tab 250mg	Ticlopidine Hydrochloride	164	162
C01BD01	Amiodarone HCl_Tab 200mg	Amiodarone Hydrochloride	3	3
C01BD07	Multaq_Tab 400mg	Dronedarone Hydrochloride	71	70
C01BC04	Flecainide Acet_Tab 50mg	Flecainide Acetate	9	9
C01BC03	Propafenone HCl_Tab 150mg	Propafenone Hydrochloride	7	7
C01BA03	Disopyramide_Cap 100mg	Disopyramide	25	25
C01BA01	Quinidine Sulf_Tab 200mg	Quinidine Sulfate	329	324
C07AA57	Sotalol HCl_Tab 40mg	Sotalol Hydrochloride	4	4
C09AA05	Ramipril_Cap 10mg	Ramipril	1	1
C09CA01	Losartan Pot_Tab 50mg	Losartan Potassium	2	2
C09XA02	Rasilez_Tab 150mg	Aliskiren	37	36
C07BB07	Bisoprolol Fumar_Tab 2.5mg	Bisoprolol Fumarate	1	1
C01AA05	Digoxin_Tab 125mcg	Digoxin	1	1
C01AA05	Amiloride HCl/Bumetanide_Tab 5mg/1mg	Amiloride HCl With Loop Diuretics	76	75
C03DA04	Eplerenone_Tab 25mg	Eplerenone	11	11
C08CA01	Amlodipine_Tab 5mg	Amlodipine	1	1
C08DB01	Tildiem LA 200_Cap 200mg	Diltiazem Hydrochloride	8	8
C01EB18	Ranexa_Tab 375mg M/R	Ranolazine	42	41
C10BX12	Atorvastatin_Tab 20mg	Atorvastatin	1	1
A10BA02	Metformin HCl_Tab 500mg	Metformin Hydrochloride	3	3
A10AD01	Ins Lantus SoloStar_100u/ml 3ml Pf Pen	Insulin Glargine	50	49
H03AA01	Levothyrox Sod_Tab 100mcg	Levothyroxine Sodium	1	1
H03BB01	Carbimazole_Tab 5mg	Carbimazole	20	20
A02BC01	Omeprazole_Cap E/C 20mg	Omeprazole	1	1
A02BA02	Ranitidine HCl_Tab 150mg	Ranitidine Hydrochloride	1	1
N06AB04	Citalopram Hydrob_Tab 20mg	Citalopram Hydrobromide	2	2
M01AE02	Naproxen_Tab 500mg	Naproxen	4	4
R03CC02	Salbutamol_Inha 100mcg (200 D) CFF	Salbutamol	2	2
R03BB04	Braltus_Pdr For Inh Cap 10mcg+Zonda Inh	Tiotropium	29	29

Missing data

Table A4 Response level data for EQ-5D-5L and mean EQ-5D-VAS at each follow-up time

	Baseline	Follow-up 1	Follow-up 2
Mobility	N (%)	N (%)	N (%)
Available data	8422 (82.2%)	6239 (60.9%)	5004 (48.8%)
I have no problems in walking about	4259 (50.6%)	3250 (52.1%)	2507 (50.1%)
I have slight problems in walking about	1948 (23.1%)	1587 (25.4%)	1349 (27.0%)
I have moderate problems in walking about	1510 (17.9%)	955 (15.3%)	755 (15.1%)
I have severe problems in walking about	630 (7.5%)	402 (6.4%)	350 (7.0%)
I am unable to walk about	75 (0.9%)	45 (0.7%)	43 (0.9%)
Self-care			
Available data	8436 (82.3%)	6246 (60.9%)	5006 (48.8%)
I have no problems washing or dressing myself	6512 (77.2%)	4822 (77.2%)	3782 (75.5%)
I have slight problems washing or dressing myself	1075 (12.7%)	889 (14.2%)	763 (15.2%)
I have moderate problems washing or dressing myself	598 (7.1%)	395 (6.3%)	325 (6.5%)
I have severe problems washing or dressing myself	187 (2.2%)	103 (1.6%)	97 (1.9%)
I am unable to wash or dress myself	64 (0.8%)	37 (0.6%)	39 (0.8%)
Usual activities			
Available data	8417 (82.1%)	6243 (60.9%)	5002 (48.8%)
I have no problems doing my usual activities	4568 (54.3%)	3508 (56.2%)	2689 (53.8%)
I have slight problems doing my usual activities	1988 (23.6%)	1540 (24.7%)	1285 (25.7%)
I have moderate problems doing my usual activities	1294 (15.4%)	859 (13.8%)	745 (14.9%)
I have severe problems doing my usual activities	430 (5.1%)	263 (4.2%)	200 (4.0%)
I am unable to do my usual activities	137 (1.6%)	73 (1.2%)	83 (1.7%)
Pain/discomfort			
Available data	8416 (82.1%)	6238 (60.9%)	5002 (48.8%)
I have no pain or discomfort	4362 (51.8%)	3454 (55.4%)	2758 (55.1%)
I have slight pain or discomfort	2266 (26.9%)	1716 (27.5%)	1403 (28.0%)
I have moderate pain or discomfort	1339 (15.9%)	784 (12.6%)	613 (12.3%)
I have severe pain or discomfort	395 (4.7%)	245 (3.9%)	201 (4.0%)
I have extreme pain or discomfort	54 (0.6%)	39 (0.6%)	27 (0.5%)
Anxiety/depression			
Available data	8412 (82.1%)	6239 (60.9%)	5001 (48.8%)
I am not anxious or depressed	4865 (57.8%)	3865 (61.9%)	3157 (63.1%)
I am slightly anxious or depressed	2141 (25.5%)	1492 (23.9%)	1219 (24.4%)
I am moderately anxious or depressed	1029 (12.2%)	682 (10.9%)	480 (9.6%)
I am severely anxious or depressed	312 (3.7%)	162 (2.6%)	116 (2.3%)
I am extremely anxious or depressed	65 (0.8%)	38 (0.6%)	29 (0.6%)
EQ-5D VAS			
Available data	7315 (71.4%)	5842 (57.0%)	4687 (45.7%)
Mean (sd)	68.6 (20.3)	70.3 (19.7)	70.8 (19.6)

Table A5 Percent missing at each time point, N=10249

	Baseline		F1		F2	
	N	%	N	%	N	%
EQ-5D-5L	1907	18.61	4031	39.33	5273	51.45
Number of ER admissions	na	na	1440	14.05	2759	26.92
Number of cardiology visits	na	na	1415	13.81	2617	25.53
Number of internal medicine/GP visits	na	na	3723	36.33	4440	43.32
Acute Coronary Syndrome	0	0	492	4.80	1880	18.34
New onset/worsening heart failure	8	0.08	296	2.89	1762	17.19
Thromboembolic events	3	0.03	494	4.82	1891	18.45
Bleeding events	3	0.03	489	4.77	1880	18.34
European Heart Rhythm Association (EHRA) classification score	1	0.01	806	7.86	2201	21.48
Clinical type of AF	177	1.73	924	9.02	2339	22.82
Vitamin K antagonists before admission	77	0.75	873	8.52	2259	22.04
NOAC user before admission	47	0.46	58	0.57	69	0.67
Antiplatelet agent use before admission	49	0.48	56	0.55	69	0.67
Age	0	0	0	0	0	0

Statistical analysis
In our study, we use fixed effects linear models to estimate the effect of symptoms and cardiovascular disease (CVD) events on our 2 primary outcomes: (1) healthcare costs; (2) health-related quality of life (HRQOL). Fixed effects Poisson models were used to estimate the relationships for three secondary outcomes: (1) Number of emergency room admissions; (2) number of cardiology visits; and (3) number of internal medicine/general practitioner (GP) visits.

The outcome model can be written as follows:

(1) $y_{it} = \beta_0 + \beta_1 x_{1_{it}} + \cdots + \beta_k x_{k_{it}} + \mu_i + \varepsilon_{i,t}$ for individual $i = 1, \ldots, N$ observed at time period $t = 1, \ldots, T$

Where

- y_{it} is the outcome of interest (e.g., healthcare costs, health-related quality of life)
- β_0 is the intercept
- $\beta_1, \ldots, \beta_k$ are the parameters
- $x_{k_{it}}$ is the k-th explanatory variable (e.g., symptoms, CVD events, age)
- μ_i is the unobservable individual-specific effect or individual heterogeneity
- $\varepsilon_{i,t}$ is the remainder disturbance

To estimate the model parameters, we used a one-way fixed-effects estimator which assumes that μ_i is a parameter that can be estimated, or that can be differenced out. The fixed effects estimator first averages equation (1) over $t = 1, \ldots, T$:

(2) $\bar{y_\imath} = \beta_0 + \beta_1 \bar{x}_{1_{it}} + \cdots + \beta_k \bar{x}_{k_{it}} + \bar{\mu}_i + \bar{\varepsilon}_{i,t}$

Subtracting (2) from (1) effectively removes the individual specific effect, μ_i. The fixed effects estimator, therefore, uses time demeaned variables:

(3) $y_{it} - \bar{y}_i = \beta_1(x_{1it} - \bar{x}_{1i}) + \beta_k(x_{kit} - \bar{x}_{ki}) + (\varepsilon_{i,t} - \bar{\varepsilon}_{i,t})$

Using Ordinary Least Squares (or Poisson) on this data produces the one way fixed effects estimates $\hat{\beta}$.

This approach contrasts with the pooled OLS (Poisson) model, which disregards μ_i as irrelevant. The pooled models combine data across individuals without accounting for individual differences. As a result, it fails to adjust for unobservable, time-invariant variables that may be correlated with the explanatory variables of interest. Ignoring unobserved individual heterogeneity μ_i may lead to severe bias of the estimated parameters, $\hat{\beta}$, if they are correlated with the individual effects.[4]

Supplemental Results

Table A6 Mean (SD) EQ-5D-5L, total healthcare costs, and number of healthcare visits by time

	Baseline Mean (SD)		F1 Mean (SD)		F2 Mean (SD)	
	Imputed	CCA	Imputed	CCA	Imputed	CCA
EQ-5D-5L (UK tariffs)	0.755 (0.003)	0.762 (0.003)	0.773 (0.003)	0.781 (0.003)	0.749 (0.005)	0.770 (0.003)
Total Healthcare costs (€)	na	na	1976 (34)	1907 (33)	1515 (33)	1351 (25)
Number of ER admissions	na	na	0.297 (0.010)	0.295 (0.009)	0.240 (0.011)	0.225 (0.008)
Number of cardiology visits	na	na	1.820 (0.023)	1.805 (0.022)	1.424 (0.020)	1.412 (0.020)
Number of internal medicine/GP visits	na	na	1.880 (0.039)	1.832 (0.041)	1.795 (0.063)	1.630 (0.044)

CCA: complete case analysis.

Table A7 Mean (standard deviation) of health-related quality of life and total healthcare costs in patients that died during follow-up compared to patients that were alive throughout the study

	HRQOL (UK tariffs) at baseline	HRQOL (UK tariffs) at F1	Health care costs (€) at baseline	Health care costs (€) at F1
Baseline	***Mean (SD)***	***Mean (SD)***	***Mean (SD)***	***Mean (SD)***
Alive at baseline and F1 N=9722	0.750 (0.003)	0.766 (0.003)	1113 (10)	2014 (34)
Alive at baseline, F1, and F2, N=9278	0.755 (0.003)	0.773 (0.003)	1112 (10)	1976 (34)
Alive at baseline but died by F1 or F2, N=971	0.628 (0.010)	n/a	1191 (40)	n/a

HRQOL: Health-related quality of life, F1: follow-up 1, F2: Follow-up 2. Estimates based on imputed samples.

Table A8 Marginal effects (95% CI) of symptoms and cardiovascular disease events on total healthcare costs, inpatient costs, outpatient costs, and medication costs in imputed vs complete case analysis

	Total cost (€)		Inpatient costs (€)		Outpatient costs (€)		Medication costs (€)	
	Imputed (N=9278)	CCA (N=7232)	Imputed (N=9278)	CCA (N=7232)	Imputed (N=9278)	CCA (N=7232)	Imputed (N=9278)	CCA (N=7232)
**Symptoms**§								
No symptoms	Reference	Reference	Reference	Reference	Reference	Reference	Reference	Reference
Mild symptoms	157 (-24, 339)	108 (-73, 289)	195* (15, 374)	104 (-72, 280)	3 (-1, 7)	2 (-3, 8)	-40* (-76, -4)	1 (-45, 47)
Severe/disabling symptoms	544*** (225, 862)	420** (106, 735)	590*** (271, 910)	435** (118, 751)	-8* (-16, -0)	-13** (-22, -4)	-39 (-79, 2)	-1 (-46, 44)
**Cardiovascular disease events**								
No cardiovascular disease events	Reference	Reference	Reference	Reference	Reference	Reference	Reference	Reference
Angina or NSTEMI+	5823*** (4757, 6889)	5526*** (4346, 6706)	5896*** (4833, 6960)	5569*** (4398, 6739)	-107*** (-125, -90)	-123*** (-142, -105)	34 (-38, 107)	80 (-2, 163)
STEMI‡	11718*** (8497, 14939)	12126*** (9507, 14744)	11674*** (8545, 14803)	12224*** (9666, 14782)	-84** (-145, -23)	-107*** (-162, -51)	128 (-253, 509)	8 (-177, 193)
New onset/worsening heart failure	3689*** (3219, 4158)	3603*** (3091, 4114)	3757*** (3290, 4224)	3687*** (3180, 4194)	-114*** (-124, -104)	-128*** (-137, -119)	46 (-15, 106)	44 (-17, 104)
Thromboembolic events	3182*** (2483, 3881)	2837*** (2239, 3434)	3256*** (2556, 3954)	2913*** (2315, 3511)	-113*** (-129, -96)	-129*** (-145, -112)	39 (-43, 120)	53 (-21, 126)
Bleeding events	3792*** (3315, 4270)	3819*** (3332, 4306)	3908*** (3439, 4377)	3937*** (3463, 4411)	-111*** (-123, -99)	-125*** (-136, -113)	-5 (-69, 59)	6 (-57, 70)

Notes: *** p<0.001, ** p<0.01, * p<0.05. §Symptoms measured using European Heart Rhythm Association (EHRA) classification score. +NSTEMI: non-ST-elevation myocardial infarction; ‡STEMI: ST segment elevation myocardial infarction. Fixed effects models adjusted for unknown time-invariant confounders and for the following observed time-variant confounders: clinical type of atrial fibrillation, age, medications used before each visit, and time period. CCA: complete case analysis.

Table A9 Marginal effects (95% CI) of symptoms and cardiovascular disease events on health-related quality of life and healthcare costs (€) in imputed vs complete case analysis

	Health-related quality of life (UK tariffs)		Healthcare costs (€)	
	Imputed (N=9278)	CCA (N=7232)	Imputed (N=9278)	CCA (N=7232)
**Symptoms§**				
No symptoms	Reference	Reference	Reference	Reference
Mild symptoms	-0.037*** (-0.048, -0.026)	-0.026 *** (-0.034, -0.017)	157 (-24, 339)	108 (-73, 289)
Severe/disabling symptoms	-0.090*** (-0.108, -0.072)	-0.081 *** (-0.096, -0.066)	544*** (225, 862)	420** (106, 735)
**Cardiovascular disease events**				
No cardiovascular disease events	Reference	Reference	Reference	Reference
Angina or NSTEMI+	-0.037* (-0.071, -0.003)	-0.029 (-0.059, 0.001)	5823*** (4757, 6889)	5526*** (4346, 6706)
STEMI‡	-0.075* (-0.144, -0.006)	-0.068* (-0.131, -0.005)	11718*** (8497, 14939)	12126*** (9507, 14744)
New onset/worsening heart failure	-0.064*** (-0.088, -0.039)	-0.062 *** (-0.086, -0.037)	3689*** (3219, 4158)	3603*** (3091, 4114)
Thromboembolic events	-0.071** (-0.115, -0.027)	-0.060* (-0.106, -0.014)	3182*** (2483, 3881)	2837*** (2239, 3434)
Bleeding events	-0.031* (-0.059, -0.003)	-0.020 (-0.052, 0.012)	3792*** (3315, 4270)	3819*** (3332, 4306)

Notes: *** p<0.001, ** p<0.01, * p<0.05. §Symptoms measured using European Heart Rhythm Association (EHRA) classification score. +NSTEMI: non-ST-elevation myocardial infarction; ‡STEMI: ST segment elevation myocardial infarction. Fixed effects models adjusted for unknown time-invariant confounders and for the following observed time-variant confounders: clinical type of atrial fibrillation, age, medications used before each visit, and time period. CCA: complete case analysis.

Sensitivity analyses

Table A10 Marginal effects (95% CI) of symptoms and cardiovascular disease events on health-related quality of life using tariffs from the United Kingdom, Spain, Germany, and Slovenia

	**Health-related quality of life UK tariffs[5]**		**Health-related quality of life Spain tariffs[6]**		**Health-related quality of life German tariffs[7]**		**Health-related quality of life Slovenian tariffs[8]**	
	Imputed (N=9278)	CCA (N=7232)	Imputed (N=9278)	CCA (N=7232)	Imputed (N=9278)	CCA (N=7232)	Imputed (N=9278)	CCA (N=7232)
**Symptoms[§]**								
No symptoms	Reference	Reference	Reference	Reference	Reference	Reference	Reference	Reference
Mild symptoms	-0.037*** (-0.048, -0.026)	-0.026 *** (-0.034,-0.017)	-0.030*** (-0.038, -0.022)	-0.022*** (-0.029, -0.014)	-0.023*** (-0.032, -0.014)	-0.014*** (-0.021, -0.007)	-0.037*** (-0.046, -0.028)	-0.032*** (-0.041, -0.023)
Severe/disabling symptoms	-0.090*** (-0.108, -0.072)	-0.081 *** (-0.096,-0.066)	-0.078*** (-0.090, -0.066)	-0.075*** (-0.088, -0.062)	-0.065*** (-0.079, -0.051)	-0.059*** (-0.072, -0.047)	-0.084*** (-0.098, -0.070)	-0.088*** (-0.103, -0.073)
**Cardiovascular disease events**								
No cardiovascular disease events	Reference	Reference	Reference	Reference	Reference	Reference	Reference	Reference
Angina or NSTEMI[+]	-0.037* (-0.071, -0.003)	-0.029 (-0.059,0.001)	-0.033* (-0.062, -0.005)	-0.018 (-0.044, 0.009)	-0.030* (-0.059, -0.001)	-0.012 (-0.038, 0.014)	-0.040* (-0.071, -0.009)	-0.031* (-0.062, -0.001)
STEMI[ǂ]	-0.075* (-0.144, -0.006)	-0.068* (-0.131,-0.005)	-0.064* (-0.118, -0.009)	-0.064* (-0.114, -0.013)	-0.058 (-0.118, 0.001)	-0.053* (-0.102, -0.004)	-0.068* (-0.136, -0.001)	-0.084** (-0.140, -0.028)
New onset/worsening heart failure	-0.064*** (-0.088, -0.039)	-0.062*** (-0.086,-0.037)	-0.048*** (-0.071, -0.024)	-0.047*** (-0.068, -0.025)	-0.042*** (-0.066, -0.018)	-0.045*** (-0.067, -0.023)	-0.061*** (-0.086, -0.035)	-0.062*** (-0.084, -0.039)
Thromboembolic events	-0.071** (-0.115, -0.027)	-0.060* (-0.106,-0.014)	-0.056** (-0.090, -0.022)	-0.059** (-0.098, -0.019)	-0.049** (-0.086, -0.012)	-0.048* (-0.090, -0.007)	-0.059** (-0.100, -0.018)	-0.065** (-0.113, -0.017)
Bleeding events	-0.031* (-0.059, -0.003)	-0.020 (-0.052,0.012)	-0.026* (-0.051, -0.000)	-0.012 (-0.039, 0.015)	-0.023 (-0.050, 0.005)	-0.012 (-0.039, 0.014)	-0.027 (-0.055, 0.001)	-0.018 (-0.049, 0.014)

Notes: *** p<0.001, ** p<0.01, * p<0.05. [§]Symptoms measured using European Heart Rhythm Association (EHRA) classification score. [+]NSTEMI: non-ST-elevation myocardial infarction; [ǂ]STEMI: ST segment elevation myocardial infarction. Fixed effects models adjusted for unknown time-invariant confounders and for the following observed time-variant confounders: clinical type of atrial fibrillation, age, medications used before each visit, and time period. CCA: complete case analysis. For Slovenia, we used the eqxw STATA command to map 5L data to 3L utilities because 5L utilities are not yet available in Slovenia.

Table A11 Marginal effects (95% CI) of symptoms and cardiovascular disease events on health-related quality of life comparing estimates when all participants included vs deceased removed

	**Health-related quality of life UK tariffs[5]**		**Health-related quality of life Spain tariffs[6]**		**Health-related quality of life German tariffs[7]**		**Health-related quality of life Slovenia tariffs[8]**	
	All participants (N=10249)	Deceased removed (N= 9278)	All participants (N=10249)	Deceased removed (N= 9278)	All participants (N=10249)	Deceased removed (N= 9278)	All participants (N=10249)	Deceased removed (N= 9278)
**Symptoms[§]**								
No symptoms	Reference	Reference	Reference	Reference	Reference	Reference	Reference	Reference
Mild symptoms	-0.036*** (-0.047,-0.026)	-0.037*** (-0.048, -0.026)	-0.030*** (-0.038,-0.021)	-0.030*** (-0.038, -0.022)	-0.023*** (-0.032,-0.014)	-0.023*** (-0.032, -0.014)	-0.037*** (-0.046,-0.027)	-0.037*** (-0.046, -0.028)
Severe/disabling symptoms	-0.088*** (-0.106,-0.069)	-0.090*** (-0.108, -0.072)	-0.076*** (-0.088,-0.065)	-0.078*** (-0.090, -0.066)	-0.063*** (-0.078,-0.049)	-0.065*** (-0.079, -0.051)	-0.082*** (-0.096,-0.069)	-0.084*** (-0.098, -0.070)
**Cardiovascular disease events**								
No cardiovascular disease events	Reference	Reference	Reference	Reference	Reference	Reference	Reference	Reference
Angina or NSTEMI[+]	-0.038* (-0.072,-0.004)	-0.037* (-0.071, -0.003)	-0.034* (-0.063,-0.006)	-0.033* (-0.062, -0.005)	-0.030* (-0.059,-0.000)	-0.030* (-0.059, -0.001)	-0.039* (-0.070,-0.008)	-0.040* (-0.071, -0.009)
STEMI[ǂ]	-0.091* (-0.163,-0.019)	-0.075* (-0.144, -0.006)	-0.073* (-0.128,-0.017)	-0.064* (-0.118, -0.009)	-0.068* (-0.128,-0.008)	-0.058 (-0.118, 0.001)	-0.081* (-0.149,-0.013)	-0.068* (-0.136, -0.001)
New onset/worsening heart failure	-0.064*** (-0.088,-0.040)	-0.064*** (-0.088, -0.039)	-0.049*** (-0.072,-0.027)	-0.048*** (-0.071, -0.024)	-0.043*** (-0.067,-0.020)	-0.042*** (-0.066, -0.018)	-0.061*** (-0.086,-0.037)	-0.061*** (-0.086, -0.035)
Thromboembolic events	-0.072** (-0.114,-0.029)	-0.071** (-0.115, -0.027)	-0.057*** (-0.090,-0.023)	-0.056** (-0.090, -0.022)	-0.050** (-0.088,-0.013)	-0.049** (-0.086, -0.012)	-0.060** (-0.101,-0.019)	-0.059** (-0.100, -0.018)
Bleeding events	-0.036** (-0.063,-0.009)	-0.031* (-0.059, -0.003)	-0.030* (-0.055,-0.005)	-0.026* (-0.051, -0.000)	-0.027 (-0.054,0.000)	-0.023 (-0.050, 0.005)	-0.032* (-0.059,-0.004)	-0.027 (-0.055, 0.001)

Notes: *** p<0.001, ** p<0.01, * p<0.05. All estimates from multiple imputed samples. [§]Symptoms measured using European Heart Rhythm Association (EHRA) classification score. [+]NSTEMI: non-ST-elevation myocardial infarction; [ǂ]STEMI: ST segment elevation myocardial infarction. Fixed effects models adjusted for unknown time-invariant confounders and for the following observed time-variant confounders: clinical type of atrial fibrillation, age, medications used before each visit, and time period. For Slovenia, we used the eqxw STATA command to map 5L data to 3L utilities because 5L utilities are not yet available in Slovenia

Table A12 Marginal effects (95% CI) of symptoms and cardiovascular disease events on healthcare costs (€) comparing costs from the United Kingdom, Spain, Germany, and Slovenia

	Healthcare costs (€) Using UK costs		Healthcare costs (€) Using Spanish costs		Healthcare costs (€) Using German costs		Healthcare costs (€) Using Slovenian costs	
	Imputed (N=9278)	CCA (N=7232)	Imputed (N=9278)	CCA (N=7232)	Imputed (N=9278)	CCA (N=7232)	Imputed (N=9278)	CCA (N=7232)
**Symptoms[§]**								
No symptoms	Reference	Reference	Reference	Reference	Reference	Reference	Reference	Reference
Mild symptoms	157 (-24, 339)	108 (-73, 289)	198* (16, 380)	114 (-69, 297)	201* (43, 359)	103 (-92, 298)	246* (17, 475)	144 (-86, 374)
Severe/disabling symptoms	544*** (225, 862)	420** (106, 735)	771*** (443,1099)	648*** (311, 985)	761*** (476,1046)	753*** (394,1112)	924*** (508, 1339)	783*** (350, 1215)
**Cardiovascular disease events**								
No cardiovascular disease events	Reference	Reference	Reference	Reference	Reference	Reference	Reference	Reference
Angina or NSTEMI[+]	5823*** (4757, 6889)	5526*** (4346, 6706)	11772*** (10762,12782)	12404*** (11472,13336)	5126*** (4452,5800)	6089*** (5242,6935)	15449*** (14134, 16764)	16442*** (15236,17648)
STEMI[‡]	11718*** (8497, 14939)	12126*** (9507, 14744)	15913*** (12510,19316)	16842*** (15313,18371)	7677*** (5770,9583)	9367*** (8063,10670)	20868*** (16420, 25317)	22260*** (20206,24314)
New onset/worsening heart failure	3689*** (3219, 4158)	3603*** (3091, 4114)	8794*** (8356,9232)	8568*** (8105,9031)	6772*** (6411,7133)	7781*** (7330,8231)	13672*** (13115, 14228)	13565*** (12974,14156)
Thromboembolic events	3182*** (2483, 3881)	2837*** (2239, 3434)	5777*** (5091,6463)	5307*** (4723,5891)	4223*** (3651,4795)	4607*** (4003,5211)	6506*** (5627, 7385)	6070*** (5319,6820)
Bleeding events	3792*** (3315, 4270)	3819*** (3332, 4306)	7480*** (6983,7977)	7272*** (6791,7753)	5895*** (5461,6329)	6713*** (6220,7207)	14353*** (13723, 4983)	14291*** (13683,14900)

Notes: *** p<0.001, ** p<0.01, * p<0.05. [§]Symptoms measured using European Heart Rhythm Association (EHRA) classification score. [+]NSTEMI: non-ST-elevation myocardial infarction; [‡]STEMI: ST segment elevation myocardial infarction. Fixed effects models adjusted for unknown time-invariant confounders and for the following observed time-variant confounders: clinical type of atrial fibrillation, age, medications used before each visit, and time period. CCA: complete case analysis.

Table A13 Marginal effects (95% CI) of symptoms and cardiovascular disease events on healthcare costs (€)) when deceased participants excluded vs included

	**Healthcare costs (€)**			
	Deceased removed N=9278		Deceased included N=10249	
**Symptoms[§]**	Marginal effects (95% CI)	p-value	Marginal effects (95% CI)	p-value
No symptoms	Reference		Reference	
Mild symptoms	157 (-24, 339)	0.090	203 (15, 392)	0.035
Severe/disabling symptoms	544 (225, 862)	0.001	492 (178, 807)	0.002
**Cardiovascular disease events**				
No cardiovascular disease events	Reference		Reference	
Angina or NSTEMI[+]	5823 (4757,6889)	p<0.001	5857 (4802, 6913)	p<0.001
STEMI[ǂ]	11718 (8497,14939)	p<0.001	9777 (6825, 12729)	p<0.001
New onset/worsening heart failure	3689 (3219,4158)	p<0.001	3822 (3389, 4254)	p<0.001
Thromboembolic events	3182 (2483,3881)	p<0.001	3415 (2733, 4097)	p<0.001
Bleeding events	3792 (3315,4270)	p<0.001	3739 (3257, 4221)	p<0.001

[§]Symptoms measured using European Heart Rhythm Association (EHRA) classification score. [+]NSTEMI: non-ST-elevation myocardial infarction; [ǂ]STEMI: ST segment elevation myocardial infarction. Fixed effects models adjusted for unknown time-invariant confounders and for the following observed time-variant confounders: clinical type of atrial fibrillation, age, medications used before each visit, and time period. Healthcare costs based on UK NHS costs reported in euros and adjusted for purchasing power parities. Based on imputed samples.

Table A14 Marginal effects (95% CI) of symptoms and cardiovascular disease events on health-related quality of life and healthcare costs (€)) comparing unweighted to IPW estimates

	Health-related quality of life (UK tariffs[5])		Healthcare costs (€)	
**Symptoms[§]**	Unweighted N=9278	IPW N=9278	Unweighted N=9278	IPW N=9278
No symptoms	Reference	Reference	Reference	Reference
Mild symptoms	-0.037*** (-0.048, -0.026)	-0.036*** (-0.047,-0.025)	157 (-24, 339)	160 (-29, 350)
Severe/disabling symptoms	-0.090*** (-0.108, -0.072)	-0.088*** (-0.107,-0.070)	544*** (225, 862)	555*** (225, 886)
**Cardiovascular disease events**				
No cardiovascular disease events	Reference	Reference	Reference	Reference
Angina or NSTEMI[+]	-0.037* (-0.071, -0.003)	-0.036* (-0.070,-0.002)	5823*** (4757,6889)	5764*** (4694,6833)
STEMI[ǂ]	-0.075* (-0.144, -0.006)	-0.077* (-0.150,-0.003)	11718*** (8497,14939)	11598*** (8270,14926)
New onset/worsening heart failure	-0.064*** (-0.088, -0.039)	-0.064*** (-0.089,-0.039)	3689*** (3219,4158)	3711*** (3229,4192)
Thromboembolic events	-0.071** (-0.115, -0.027)	-0.078** (-0.127,-0.030)	3182*** (2483,3881)	3162*** (2420,3903)
Bleeding events	-0.031* (-0.059, -0.003)	-0.033* (-0.062,-0.004)	3792*** (3315,4270)	3773*** (3289,4256)

Notes: *** p<0.001, ** p<0.01, * p<0.05. IPW: inverse probability weighting, these were constructed using a logistic regression model which predicted the probability of being alive conditional on the baseline characteristics listed in Table 1. Each participant's weight was the inverse of this predicted probability. These weights were applied to the FE regressions. [§]Symptoms measured using European Heart Rhythm Association (EHRA) classification score. [+]NSTEMI: non-ST-elevation myocardial infarction; [ǂ]STEMI: ST segment elevation myocardial infarction. Fixed effects models adjusted for unknown time-invariant confounders and for the following observed time-variant confounders: clinical type of atrial fibrillation, age, medications used before each visit, and time period. Based on imputed samples.

Table A15 Incidence rate ratios (95% CI) of symptoms and cardiovascular disease events on healthcare visits comparing unweighted to IPW estimates

	No. of ER admissions		No. of cardiology visits		No. of internal medicine/GP visits	
**Symptoms[§]**	Unweighted N=9278	IPW N=9278	Unweighted N=9278	IPW N=9278	Unweighted N=9278	IPW N=9278
No symptoms	Reference	Reference	Reference	Reference	Reference	Reference
Mild symptoms	1.51*** (1.30, 1.77)	1.50*** (1.29, 1.73)	1.13*** (1.06, 1.20)	1.13*** (1.06, 1.20)	1.19*** (1.09, 1.29)	1.18*** (1.09, 1.28)
Severe/disabling symptoms	2.14*** (1.79, 2.56)	2.09*** (1.75, 2.48)	1.23*** (1.12, 1.34)	1.23*** (1.13, 1.34)	1.20** (1.05, 1.38)	1.20** (1.05, 1.37)
**Cardiovascular disease events**						
No cardiovascular disease events	Reference	Reference	Reference	Reference	Reference	Reference
Angina or NSTEMI[+]	2.49*** (1.78, 3.49)	2.52*** (1.82, 3.47)	1.05 (0.88, 1.25)	1.05 (0.89, 1.24)	0.92 (0.70, 1.21)	0.91 (0.69, 1.19)
STEMI[ǂ]	1.72 (0.78, 3.77)	1.75 (0.83, 3.70)	1.03 (0.66, 1.61)	1.04 (0.68, 1.58)	1.02 (0.33, 3.14)	0.97 (0.34, 2.82)
New onset/worsening heart failure	1.71*** (1.38, 2.11)	1.77*** (1.45, 2.15)	1.32*** (1.19, 1.47)	1.33*** (1.20, 1.46)	1.11 (0.94, 1.31)	1.09 (0.93, 1.28)
Thromboembolic events	2.05*** (1.38, 3.04)	2.01*** (1.37, 2.95)	1.21* (1.01, 1.46)	1.21* (1.00, 1.45)	1.44* (1.11, 1.88)	1.42* (1.08, 1.87)
Bleeding events	2.48*** (1.82, 3.37)	2.54*** (1.89, 3.40)	1.13 (0.99, 1.29)	1.13 (0.99, 1.28)	1.33** (1.07, 1.65)	1.33** (1.07, 1.64)

Notes: *** p<0.001, ** p<0.01, * p<0.05. IPW: inverse probability weighting, these were constructed using a logistic regression model which predicted the probability of being alive conditional on the baseline characteristics listed in Table 1. Each participant's weight was the inverse of this predicted probability. These weights were applied to the FE regressions. [§]Symptoms measured using European Heart Rhythm Association (EHRA) classification score. [+]NSTEMI: non-ST-elevation myocardial infarction; [ǂ]STEMI: ST segment elevation myocardial infarction. Fixed effects Poisson models adjusted for unknown time-invariant confounders and for the following observed time-variant confounders: clinical type of atrial fibrillation, age, medications used before each visit, and time period. Based on imputed samples.

Table A16 Incidence rate ratios (95% CI) of number of ER admissions, cardiology visits, internal medicine/GP visits by region

	No. of ER admissions	No. of cardiology visits	No. of internal medicine/GP visits
**Symptoms§**	IRR (95% CI)	IRR (95% CI)	IRR (95% CI)
Mild symptoms x Western EU	Reference	Reference	Reference
Mild symptoms x Eastern EU	0.78 (0.46, 1.35)	0.89 (0.75, 1.06)	0.92 (0.76, 1.12)
Mild symptoms x Northern EU	0.74 (0.46, 1.21)	0.96 (0.80,1.16)	1.11 (0.89, 1.40)
Mild symptoms x Southern EU	0.78 (0.53, 1.17)	0.93 (0.80, 1.08)	1.09 (0.87, 1.36)
Severe/disabling symptoms x Western EU	Reference	Reference	Reference
Severe/disabling symptoms x Eastern EU	2.12 (1.18, 3.80)	1.20 (0.93, 1.55)	1.10 (0.83, 1.46)
Severe/disabling symptoms x Northern EU	1.73 (0.97, 3.09)	1.51 (1.14, 1.99)	0.90 (0.65, 1.25)
Severe/disabling symptoms x Southern EU	1.66 (1.02, 2.70)	1.08 (0.85, 1.37)	1.33 (0.89, 1.98)
**Cardiovascular disease events**			
Acute coronary syndromes x Western EU	Reference	Reference	Reference
Acute coronary syndromes x Eastern EU	0.93 (0.28, 3.12)	0.87 (0.55, 1.36)	1.06 (0.60, 1.86)
Acute coronary syndromes x Northern EU	0.71 (0.25, 2.02)	1.20 (0.66, 2.16)	0.71 (0.31, 1.62)
Acute coronary syndromes x Southern EU	0.43 (0.18, 1.02)	1.00 (0.68, 1.48)	1.41 (0.71, 2.82)
New onset/worsening heart failure x Western EU	Reference	Reference	Reference
New onset/worsening heart failure x Eastern EU	0.60 (0.29, 1.25)	0.92 (0.68, 1.26)	1.13 (0.79, 1.62)
New onset/worsening heart failure x Northern EU	0.89 (0.47, 1.70)	1.16 (0.84, 1.60)	1.03 (0.66, 1.58)
New onset/worsening heart failure x Southern EU	0.58 (0.35, 0.95)	1.05 (0.82, 1.35)	1.46 (1.01, 2.12)
Thromboembolic events x Western EU	Reference	Reference	Reference
Thromboembolic events x Eastern EU	1.26 (0.21, 7.78)	0.66 (0.38, 1.15)	1.11 (0.57, 2.14)
Thromboembolic events x Northern EU	3.61 (0.89, 14.61)	1.22 (0.71, 2.08)	1.03 (0.55, 1.91)
Thromboembolic events x Southern EU	0.73 (0.31, 1.70)	0.96 (0.59, 1.56)	1.87 (0.95, 3.68)
Bleeding events x Western EU	Reference	Reference	Reference
Bleeding events x Eastern EU	1.54 (0.43, 5.51)	0.95 (0.60, 1.50)	0.65 (0.40, 1.05)
Bleeding events x Northern EU	1.70 (0.63, 4.64)	0.85 (0.57, 1.29)	1.45 (0.92, 2.29)
Bleeding events x Southern EU	1.39 (0.73, 2.63)	0.78 (0.56, 1.09)	0.65 (0.40, 1.05)

Notes: Acute coronary syndromes includes Angina/NSTEMI and STEMI. §Symptoms measured using European Heart Rhythm Association (EHRA) classification score. Fixed effects models with interaction terms for symptoms and cardiovascular disease events with region. Models adjusted for unknown time-invariant confounders and for the following observed time-variant confounders: clinical type of atrial fibrillation, age, medications used before each visit, and time period. Western EU is the reference category. Northern EU countries: Denmark, Estonia, Latvia, Norway and the United Kingdom (UK); Western EU countries: Belgium, France, Germany, Netherlands, Switzerland); Eastern EU countries: Bulgaria, Czech Republic, Georgia, Kazakhstan, Kyrgyzstan, Poland, Romania and Russia; Southern EU countries: Albania, North Macedonia, Italy, Malta, Montenegro, Portugal, Serbia, Spain and Turkey.

Table A17 Incidence rate ratios (95% CI) of number of ER admissions, cardiology visits, internal medicine/GP visits by sex

	No. of ER admissions	No. of cardiology visits	No. of internal medicine/GP visits
**Symptoms§**	IRR (95% CI)	IRR (95% CI)	IRR (95% CI)
Mild symptoms x Men	Reference	Reference	Reference
Mild symptoms x Women	1.15 (0.85, 1.56)	1.01 (0.90, 1.14)	0.93 (0.80, 1.08)
Severe/disabling symptoms x Men	Reference	Reference	Reference
Severe/disabling symptoms x Women	1.21 (0.83, 1.75)	1.03 (0.87, 1.23)	1.03 (0.82, 1.29)
**Cardiovascular disease events**			
Acute coronary syndromes x Men	Reference	Reference	Reference
Acute coronary syndromes x Women	0.68 (0.37, 1.25)	1.16 (0.82, 1.63)	1.22 (0.78, 1.90)
New onset/worsening heart failure x Men	Reference	Reference	Reference
New onset/worsening heart failure x Women	0.63 (0.42, 0.93)	1.03 (0.84, 1.27)	1.33 (1.00, 1.77)
Thromboembolic events x Men	Reference	Reference	Reference
Thromboembolic events x Women	1.18 (0.57, 2.44)	1.25 (0.86, 1.81)	1.12 (0.68, 1.83)
Bleeding events x Men	Reference	Reference	Reference
Bleeding events x Women	0.64 (0.37, 1.13)	0.94 (0.72, 1.24)	1.26 (0.88, 1.81)

Notes: Symptoms measured using European Heart Rhythm Association (EHRA) classification score. Acute coronary syndromes includes Angina/NSTEMI and STEMI. Fixed effects models with interaction terms for symptoms and cardiovascular disease events with biological sex. Models adjusted for unknown time-invariant confounders and for the following observed time-variant confounders: clinical type of atrial fibrillation, age, medications used before each visit, and time period. Men is the reference category.

STROBE Statement—Checklist of items that should be included in reports of *cohort studies*

	Item No	Recommendation	Location in manuscript
Title and abstract	1	(*a*) Indicate the study's design with a commonly used term in the title or the abstract	Pages 1,2, under 'Title' and 'Abstract'
		(*b*) Provide in the abstract an informative and balanced summary of what was done and what was found	Page 2, under 'Abstract'
Introduction			
Background/rationale	2	Explain the scientific background and rationale for the investigation being reported	Page 3, under 'Introduction'
Objectives	3	State specific objectives, including any prespecified hypotheses	Page 3, under 'Introduction'
Methods			
Study design	4	Present key elements of study design early in the paper	Page 4, under 'Study design and procedures'
Setting	5	Describe the setting, locations, and relevant dates, including periods of recruitment, exposure, follow-up, and data collection	Pages 4, 5, 6 under 'Methods', page 7 of 'Results, and page 2 of 'Supplemental appendix'
Participants	6	(*a*) Give the eligibility criteria, and the sources and methods of selection of participants. Describe methods of follow-up	Page 4 under 'Study design and procedures'
		(*b*) For matched studies, give matching criteria and number of exposed and unexposed	Not applicable
Variables	7	Clearly define all outcomes, exposures, predictors, potential confounders, and effect modifiers. Give diagnostic criteria, if applicable	Pages 4,5,6 under 'Methods'
Data sources/ measurement	8*	For each variable of interest, give sources of data and details of methods of assessment (measurement). Describe comparability of assessment methods if there is more than one group	Pages 4,5,6 under 'Methods'
Bias	9	Describe any efforts to address potential sources of bias	Page 5,6 under Methods (i.e., 'Outcomes' and 'Missing data')
Study size	10	Explain how the study size was arrived at	Page 4, under 'Study design and procedures, and page 7 under 'Results'
Quantitative variables	11	Explain how quantitative variables were handled in the analyses. If applicable, describe which groupings were chosen and why	Page 6, under 'Statistical analysis'

Statistical methods	12	(*a*) Describe all statistical methods, including those used to control for confounding	Page 6 under ‘Statistical analysis’ and page 3-9 of ‘Supplemental appendix’.
		(*b*) Describe any methods used to examine subgroups and interactions	Page 6 under ‘Statistical analysis’
		(*c*) Explain how missing data were addressed	Page 6 under ‘Missing data’ and page 7,8 of ‘Supplemental appendix’
		(*d*) If applicable, explain how loss to follow-up was addressed	Not applicable
		(*e*) Describe any sensitivity analyses	Page 5 under ‘Outcomes’
Results			
Participants	13*	(a) Report numbers of individuals at each stage of study—eg numbers potentially eligible, examined for eligibility, confirmed eligible, included in the study, completing follow-up, and analysed	Page 8 under ‘Results’,
		(b) Give reasons for non-participation at each stage	Not applicable
		(c) Consider use of a flow diagram	Not applicable
Descriptive data	14*	(a) Give characteristics of study participants (eg demographic, clinical, social) and information on exposures and potential confounders	Page 8 under ‘Results’ and Table 1
		(b) Indicate number of participants with missing data for each variable of interest	Table 1 and Supplemental Table A4 & A5
		(c) Summarise follow-up time (eg, average and total amount)	Page 4,6 under ‘Methods’
Outcome data	15*	Report numbers of outcome events or summary measures over time	Supplemental Table A5
Main results	16	(*a*) Give unadjusted estimates and, if applicable, confounder-adjusted estimates and their precision (eg, 95% confidence interval). Make clear which confounders were adjusted for and why they were included	Tables 2,3,4 and Figures 1,2
		(*b*) Report category boundaries when continuous variables were categorized	Not applicable, continuous variables were not transformed into categories
		(*c*) If relevant, consider translating estimates of relative risk into absolute risk for a meaningful time period	Not relevant

Other analyses	17	Report other analyses done—eg analyses of subgroups and interactions, and sensitivity analyses	Page 10, under 'Sensitivity analyses' and pages 10-21 of 'Supplemental appendix'.
Discussion			
Key results	18	Summarise key results with reference to study objectives	Page 11 under Discussion
Limitations	19	Discuss limitations of the study, taking into account sources of potential bias or imprecision. Discuss both direction and magnitude of any potential bias	Page 12, 13 under 'Discussion' sub-section 'Limitations'
Interpretation	20	Give a cautious overall interpretation of results considering objectives, limitations, multiplicity of analyses, results from similar studies, and other relevant evidence	Page 11, 12, 13, 14 under 'Discussion'
Generalisability	21	Discuss the generalisability (external validity) of the study results	Page 11, 12, 13, 14
Other information			
Funding	22	Give the source of funding and the role of the funders for the present study and, if applicable, for the original study on which the present article is based	Page 15

*Give information separately for exposed and unexposed groups.

References

1. HRG4+ 2018/19 Reference Costs Grouper. *NHS Digital*. https://digital.nhs.uk/services/national-casemix-office/downloads-groupers-and-tools/costing---hrg4-2018-19-reference-costs-grouper (4 August 2023)

2. Prescription Cost Analysis - England, 2018 [PAS]. *NDRS*. https://digital.nhs.uk/data-and-information/publications/statistical/prescription-cost-analysis/2018 (2 August 2023)

3. NHS England » 2018/19 National Cost Collection Data Publicationhttps://www.england.nhs.uk/publication/2018-19-national-cost-collection-data-publication/ (4 August 2023)

4. Wooldridge, Jeffrey. *Econometric Analysis of Cross Section and Panel Data*. 2nd edition. MIT Press.

5. Hernández Alava M, Pudney S, Wailoo A. Estimating the Relationship Between EQ-5D-5L and EQ-5D-3L: Results from a UK Population Study. *PharmacoEconomics* 2023;**41**:199–207.

6. Ramos-Goñi JM, Craig BM, Oppe M, Ramallo-Fariña Y, Pinto-Prades JL, Luo N, Rivero-Arias O. Handling Data Quality Issues to Estimate the Spanish EQ-5D-5L Value Set Using a Hybrid Interval Regression Approach. *Value Health* 2018;**21**:596–604.

7. Ludwig K, Graf Von Der Schulenburg J-M, Greiner W. German Value Set for the EQ-5D-5L. *PharmacoEconomics* 2018;**36**:663–674.

8. Prevolnik Rupel V, Srakar A, Rand K. Valuation of EQ-5D-3l Health States in Slovenia: VAS based and TTO based Value Sets. *Zdr Varst* 2020;**59**:8–17.
